# Requirement of *CHROMOMETHYLASE3* for somatic inheritance of the spontaneous tomato epimutation *Colourless non-ripening*

**DOI:** 10.1038/srep09192

**Published:** 2015-03-17

**Authors:** Weiwei Chen, Junhua Kong, Cheng Qin, Sheng Yu, Jinjuan Tan, Yun-ru Chen, Chaoqun Wu, Hui Wang, Yan Shi, Chunyang Li, Bin Li, Pengcheng Zhang, Ying Wang, Tongfei Lai, Zhiming Yu, Xian Zhang, Nongnong Shi, Huizhong Wang, Toba Osman, Yule Liu, Kenneth Manning, Stephen Jackson, Dominique Rolin, Silin Zhong, Graham B. Seymour, Philippe Gallusci, Yiguo Hong

**Affiliations:** 1 Research Centre for Plant RNA Signalling, College of Life and Environmental Sciences, Hangzhou Normal University, Hangzhou 310036, China; 2 State Key Laboratory of Agrobiotechnology, School of Life Sciences, The Chinese University of Hong Kong, Hong Kong; 3 School of Life Sciences, University of Warwick, Coventry CV4 7AL, UK; 4 MOE Key Laboratory of Bioinformatics, Centre for Plant Biology, School of Life Sciences, Tsinghua University, Beijing 100084, China; 5 UMR Fruit Biology and Pathology, Bordeaux University, INRA, Villenave d′Ornon 33883, France; 6 Plant and Crop Science Division, School of Biosciences, University of Nottingham, Loughborough, Leics LE12 5RD, UK

## Abstract

Naturally-occurring epimutants are rare and have mainly been described in plants. However how these mutants maintain their epigenetic marks and how they are inherited remain unknown. Here we report that *CHROMOMETHYLASE3* (*SlCMT3*) and other methyltransferases are required for maintenance of a spontaneous epimutation and its cognate Colourless non-ripening (*Cnr*) phenotype in tomato. We screened a series of DNA methylation-related genes that could rescue the hypermethylated *Cnr* mutant. Silencing of the developmentally-regulated *SlCMT3* gene results in increased expression of *LeSPL-CNR*, the gene encodes the SBP-box transcription factor residing at the *Cnr* locus and triggers *Cnr* fruits to ripen normally. Expression of other key ripening-genes was also up-regulated. Targeted and whole-genome bisulfite sequencing showed that the induced ripening of *Cnr* fruits is associated with reduction of methylation at CHG sites in a 286-bp region of the *LeSPL-CNR* promoter, and a decrease of DNA methylation in differentially-methylated regions associated with the LeMADS-RIN binding sites. Our results indicate that there is likely a concerted effect of different methyltransferases at the *Cnr* locus and the plant-specific *SlCMT3* is essential for sustaining *Cnr* epi-allele. Maintenance of DNA methylation dynamics is critical for the somatic stability of *Cnr* epimutation and for the inheritance of tomato non-ripening phenotype.

Spontaneous epimutations can result from heritable changes in DNA methylation without alteration in the underlying sequence, but these changes can influence gene expression and associated phenotypes^1^,^2^,^3^,^4^,^5^. Indeed epimutations can affect inbred traits in plants and animals^6^,^7^,^8^,^9^,^10^,^11^,^12^,^13^,^14^. However natural epigenetic variations are rare and little is known about how spontaneous epimutations retain their heritable stability^1^,^2^,^3^,^4^,^5^. In plants, methylation occurs at cytosines in CG, CHG and CHH contexts (where H = A, T, C) through the combined enzymatic activity of DOMAINS REARRANGED METHYLTRANSFERASEs (DRMs), METHYLTRANSFERASE1 (MET1) and the plant specific CHROMOMETHYLASEs (CMTs)^15^,^16^. These enzymes are required for RNA-directed DNA methylation (RdDM) and methylation maintenance. In *Arabidopsis*, DRM2 catalyses *de novo* methylation in all sequence contexts and CMT2 is involved in non-symmetrical methylation while MET1, CMT3 and DRM2 participate in methylation maintenance at the CG, CHG and CHH sites, respectively^15^,^16^.

The tomato *Colourless non-ripening* (*Cnr*) is one of the best characterized naturally occurring epimutants^3^. *Cnr* differs from structural epi-variants such as *CmWIP*, *FWA*, *FOLT1* and *SP11*^17^,^18^,^19^,^20^ in *Arabidopsis*, melon and *Brassica*, of which the epigenetic changes are either induced by transposon or trans-acting small RNAs, or genetic non-ripening mutants such as tomato *rin*, *ripening-inhibitor*^21^. *Cnr* contains eighteen hypermethylated cytosines in a 286-bp region of the *LeSPL-CNR* promoter at the *Cnr* locus and the *Cnr* epimutation and phenotype are very stable^3^. We only observed four *Cnr* fruits with revertant sectors showing red stripes out of thousands of fruits grown over more than twenty years. In this paper, using the spontaneous *Cnr* epimutant together with VIGS-based gene functional screening, targeted and whole-genome DNA methylation profiling and qRT-PCR assay, we investigate the mechanism responsible for somatic inheritance of *Cnr*. We unravel that *SlCMT3* silencing results in reduction of DNA methylation and leads to *Cnr*-to-ripening reversion in tomato. Our results demonstrate that *SlCMT3*, possibly along with other key components including *SlCMT2*, *SlDRM7* and *SlMET1* in the RdDM and methylation maintenance pathways, is required to maintain the *Cnr* epi-allele, and *CMT3* possesses an important role in epigenetic regulation of structural genes such as transcription factors in addition to its role in maintaining the methylation of repetitive DNA and transposon-related sequences.

## Results

### Silencing of DNA methylation-associated genes affects *Cnr* fruit ripening

**Figure 1:**
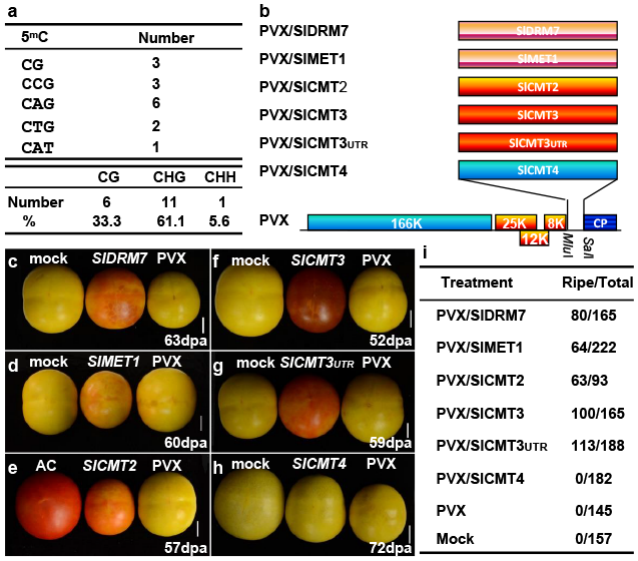
*SlDMT* silencing causes *Cnr* epimutant to ripening. (a), Context, number and percentage of the hypermethylated cytosines (5^m^C) in the 286-bp *LeSPL-CNR* promoter region. (b), Diagram of VIGS vectors PVX/SlDRM7, PVX/SlMET1, PVX/SlCMT2, PVX/SlCMT3, PVX/SlCMT3UTR and PVX/SlCMT4. (c–h), Ripening in *Cnr* fruits, assessed by red colour as compared to wild-type fruits (AC, (e)). No ripening was observed in fruits mock-inoculated (mock), inoculated with PVX or PVX/SlCMT4 (h). Photographs were taken at the indicated day post-anthesis (dpa). Bar = 1 cm. (i), Number of ripening fruits out of total number of inoculated fruits from at least two independent experiments.

*Cnr* phenotype could be recreated in normal fruits by repression of *LeSPL-CNR*^3^,^22^ or by increasing methylation level in the 286-bp region^23^ (Supplementary Fig. 1), demonstrating that hypermethylation causes the phenotype. The eighteen hypermethylated cytosines in a 286-bp region of the *LeSPL-CNR* promoter are thought to be responsible for the non-ripening phenotype (Fig. 1a). To uncover the mechanism guarding the stability of the *Cnr* epi-allele, we used *Potato virus X* (PVX)-based VIGS^3^,^22^ to silence a range of DNA methylation-associated genes including *SlDRM7*, *SlMET1*, *SlCMT2*, *SlCMT3* and *SlCMT4*^24^ (Fig. 1b). These genes were selected based on sequence homology to the well-characterized Arabidopsis DNA-methyltransferases (DMTs; Supplementary Fig. 2). Specific cDNA fragments corresponding to each of the *SlDMT* genes were cloned into the PVX-based VIGS vector (Fig. 1b). It is worthwhile noting that nucleotide similarities among sequences of VIGS inducers are mostly around 30% or lower (Supplementary Table 1). Considering the requirement of perfect complementarity between silencing inducer and target sequences for small RNA (siRNA and microRNA)-mediated silencing in plants, we expect that these constructs including PVX/SlCMT2 and PVX/SlCMT3 should target their intended genes for gene-specific VIGS.

Indeed, *Cnr* fruits undergoing VIGS of *SlDRM7*, *SlMET1*, *SlCMT2* and *SlCMT3* ripened to various degrees (Fig. 1c–e, Supplementary Fig. 3a–n). Particularly VIGS of *SlCMT3* by PVX/SlCMT3, targeting the coding region of *SlCMT3* mRNA, caused *Cnr* fruits to reach the stage of losing chlorophyll (equivalent to breaker) approximately 4 days earlier than *Cnr* fruits mock-inoculated with TE buffer or injected with PVX (Supplementary Fig. 4). *SlCMT3*-silenced fruits continued to ripen almost completely (Fig. 1f, Supplementary Fig. 5a–h). PVX/SlCMT3_UTR_ targeting the 3′-UTR of *SlCMT3* mRNA could also trigger *Cnr* fruit ripening (Fig. 1g, Supplementary Fig. 6a–i). However, not all *CMT* genes are necessary for maintenance of *Cnr* since *SlCMT4* silencing had no effect on ripening (Fig. 1h, Supplementary Fig. 3o), further demonstrating that the observed ripening phenotypes were resulted from gene-specific VIGS by specific *SlDMT* constructs (Fig. 1b–i).

More than 60% of fruits at 5–15 days post anthesis were injected with PVX/SlCMT3, PVX/SlCMT3_UTR_ or PVX/SlCMT2 developed ripening phenotype. Only approximately 29% and 48% of fruits treated with PVX/SlMET1 or PVX/SlDRM7 appeared ripening. There was no ripening of *Cnr* fruits treated with PVX/SlCMT4, empty VIGS vector PVX, or mock-inoculated (Fig. 1i). It is worthwhile noting that no ripening was observed in *rin* fruits injected with PVX/SlCMT3 (Supplementary Fig. 3p). Taken together, our results demonstrate that functional *SlDMTs* in the RdDM and methylation maintenance pathways are required for maintain the somatic stability of the non-ripening *Cnr* phenotype in the natural epimutant.

### Developmentally regulated *SlCMT3* is likely the key modulator for maintaining the *Cnr* epi-allele

In *Arabidopsis*, *CMT* genes are predominantly associated with maintenance of cytosine methylation in transposable elements^12^,^13^,^15^. It is therefore surprising that silencing of *SlCMT2* and *SlCMT3* (a close relative of *Arabidopsis CMT3*) should rescue *Cnr* ripening. It is also intriguing that *SlCMT3* silencing had a greater effect on reverting the *Cnr* phenotype than silencing of *SlDRM7* (a homologue of the Arabidopsis *de novo* methyltransferase *DRM2*) or other *SlDMTs* (Fig. 1). These phenotypic differences may be due to variations in VIGS efficiencies, although this is unlikely because the PVX system is highly effective at silencing genes in tomato^3^,^22^. Alternatively, our results may suggest that *SlCMT3* plays a more prominent role in maintaining epi-alleles such as *Cnr* than *SlDRM7* and other *SlDMTs*. This is consistent with a high frequency of CHG hypermethylation in the *LeSPL-CNR* epimutated-region (Fig. 1a), the maintenance of which mainly requires functional *SlCMT3*^16^. We interpret these data to mean that *SlCMT3* is probably one of the key genetic regulators underlying the inheritable maintenance of *Cnr* epimutation.

**Figure 2:**
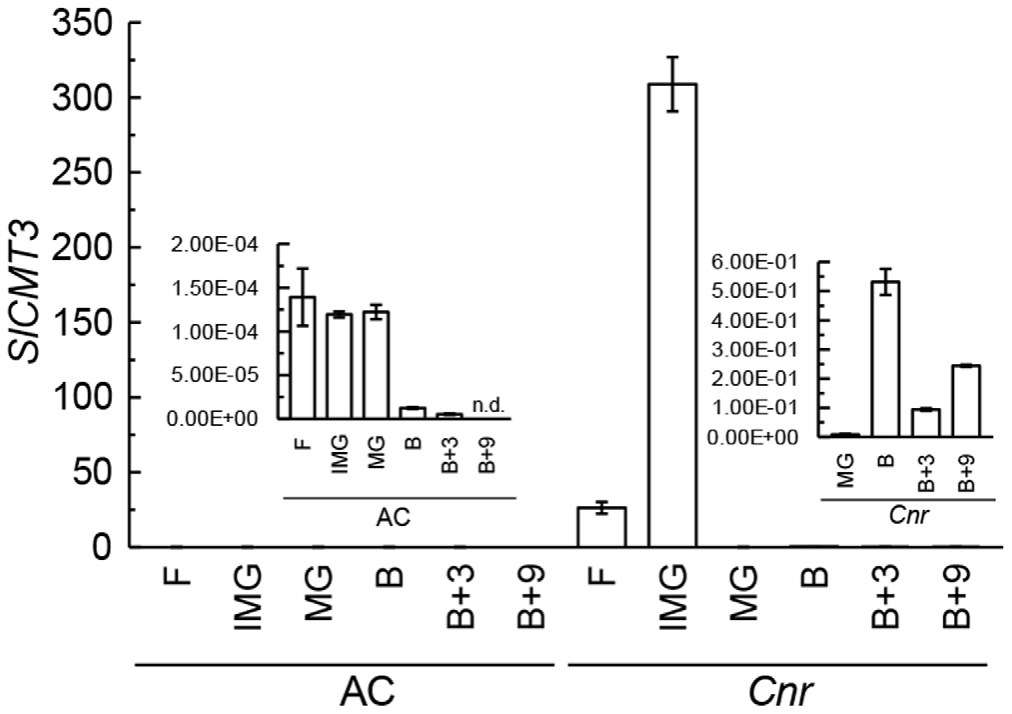
Developmental regulation of *SlCMT3* expression. Relative levels of *SlCMT3* mRNA in fully-opened flowers (F) and pericarps from wild-type (AC) and *Cnr* epimutant fruits at immature green (IMG), mature green (MG), breaker (B), breaker + three days (B+3) and breaker + nine days (B+9) stages. The inset-figures have different y-axis scales to show the low levels of *SlCMT3* mRNA at different ripening stages of in the AC and *Cnr* fruits. These values are dwarfed by the exceptionally high levels of expression of *SlCMT3* in IMG *Cnr* fruit.

This hypothesis is supported by the fact that *SlCMT3* expression is subject to developmental regulation. Expression of *SlCMT3* changed dramatically in developing *Cnr* fruits, being extremely high at the immature stage then declining in mature green fruits (Fig. 2). The levels of *SlCMT3* expression in immature *Cnr* fruits are so high that they dwarf those at all other stages of fruit development in normal and *Cnr* fruits (inset panels, Fig. 2). The *SlCMT3* transcripts were again up-regulated in fruits at breaker before declining to lower levels in later stages. Expression of *SlCMT3* in normal fruits was highest in green stages, but significantly lower than in immature *Cnr* fruits, and was down-regulated at breaker stage (Fig. 2). The prominent quantitative differences in expression of *SlCMT3* between wild-type and *Cnr* fruits suggest that high level expression of *SlCMT3* may be associated with the maintenance of the *Cnr* epi-status.

### Silencing of *SlCMT3* enhances *LeSPL-CNR* and other key ripening TF gene expression

**Figure 3:**
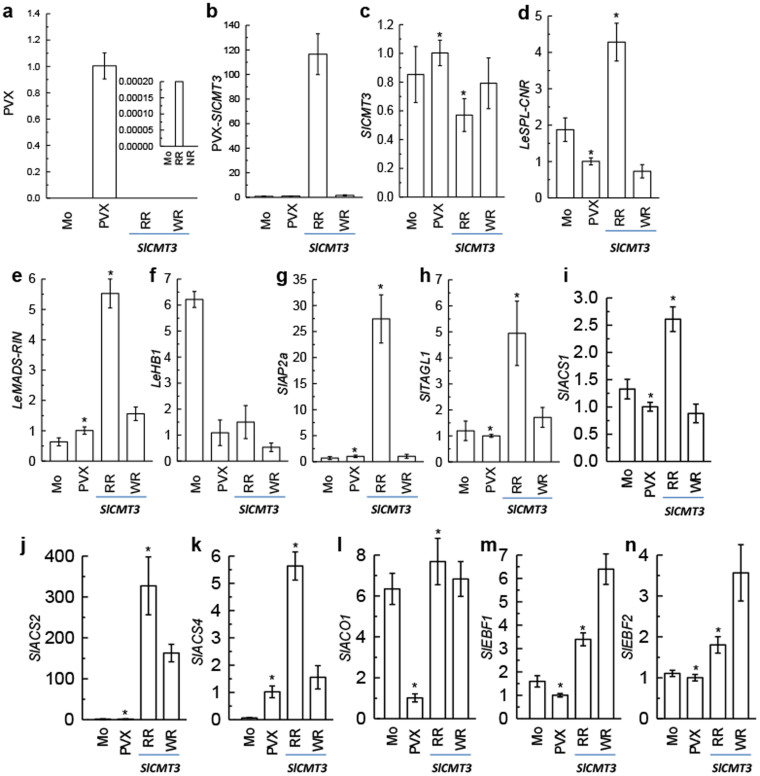
*SlCMT3* affects expression of *LeSPL-CNR* and ripening genes. (a), PVX RNA. (b), Silencing trigger RNA (PVX-SlCMT3). (c–n), Endogenous *SlCMT3*, *LeSPL-CNR*, *LeMADS-RIN*, *LeHB1*, *SlAP2a*, *SlTAGL1*, *SlACS1*, *SlACS2*, *SlACS4*, *SlACO1*, *SlEBF1* and *SlEBF2* mRNAs in non-ripening fruits mock-inoculated (Mo), inoculated with PVX, or in red-ripening (RR) and weak-ripening (WR) sectors of *Cnr* fruits inoculated with PVX/SlCMT3 (SlCMT3) at 31 days post inoculation. The inset-figure in (a) shows a low level of PVX RNA. Asterisk (*) indicates statistical significance (p < 0.001) by Student's *t*-tests between the *SlCMT3*-silenced and PVX control samples.

To dissect the mechanism by which *SlCMT3* repression causes the reversion of the *Cnr* to ripening, we analyzed whether *SlCMT3* silencing affects expression of *LeSPL-CNR* and other key ripening transcription factor (TF) genes including *LeMADS-RIN*, *LeHB1*, *SlAP2a* and *SlTAGL1*^22^,^25^,^26^,^27^. Viral RNA declined dramatically in PVX/SlCMT3-injected fruits (Fig. 3a) and the silencing trigger *SlCMT3* RNA was detected (Fig. 3b). Endogenous *SlCMT3* mRNA in ripening pericarps was significantly reduced although only a moderate decrease was observed in the weakly ripe tissues of the same fruits (Fig. 3c, Supplementary Fig. 7a). In contrast with the reduction of *SlCMT3* mRNA in silenced fruits, *LeSPL-CNR* was up-regulated when compared to levels in the control (Fig. 3d, Supplementary Fig. 7b). *LeMADS-RIN*, *SlAP2a* and *SlTAGL1* were also up-regulated, although *LeHB1* expression was not significantly affected (Fig. 3e–h, Supplementary Fig. 7c–f). It should be noted that all TFs tested are known to be developmentally regulated in normal and *Cnr* fruits, although their expression levels differ and are generally much lower in *Cnr*^3^,^22^,^25^,^26^,^27^ (Supplementary Fig. 7g–h). These results demonstrate that *Cnr*-to-ripening reversion by *SlCMT3* silencing is inversely correlated not only to the expression of *LeSPL-CNR*, but also to that of other ripening-associated TF genes. However, how VIGS of *SlCMT3* influences expression of additional ripening TF genes remains to be elucidated. It is possible that such an impact could be a secondary effect of ripening or the change of the *LeSPL-CNR* expression, or/and is due to altered methylation of promoters of these TF genes.

### Silencing of *SlCMT3* enhances expression of genes involved in the biosynthesis and signal transduction of the ripening hormone ethylene

We also examined the expression of ethylene biosynthesis genes *SlACS1*, *SlACS2*, *SlACS4* and *SlACO1*, and two ethylene signal transduction genes *SlEBF1* and *SlEBF2*^24^ during ripening of *Cnr* fruits. Consistent with up-regulation of ripening-associated TF gene expression, these ripening hormone-related genes were all found to be up-regulated in the ripe pericarp tissues in which *SlCMT3* was silenced (Fig. 3i–m, Supplementary Fig. 8a–f). Indeed TFs such as LeMADS-RIN are known to regulate the expression of ethylene biosynthetic genes^25^. It is also possible that *SlCMT3* is involved in the epigenetic regulation of these genes because levels of DNA methylation in their promoter regions in *SlCMT3*-silenced fruits were reduced, or that their up-regulation is the direct or indirect down-stream effect of *LeSPL-CNR*.

### Silencing of *SlCMT3* reduces cytosine methylation in the epimutated region of the *LeSPL-CNR* promoter

**Figure 4:**
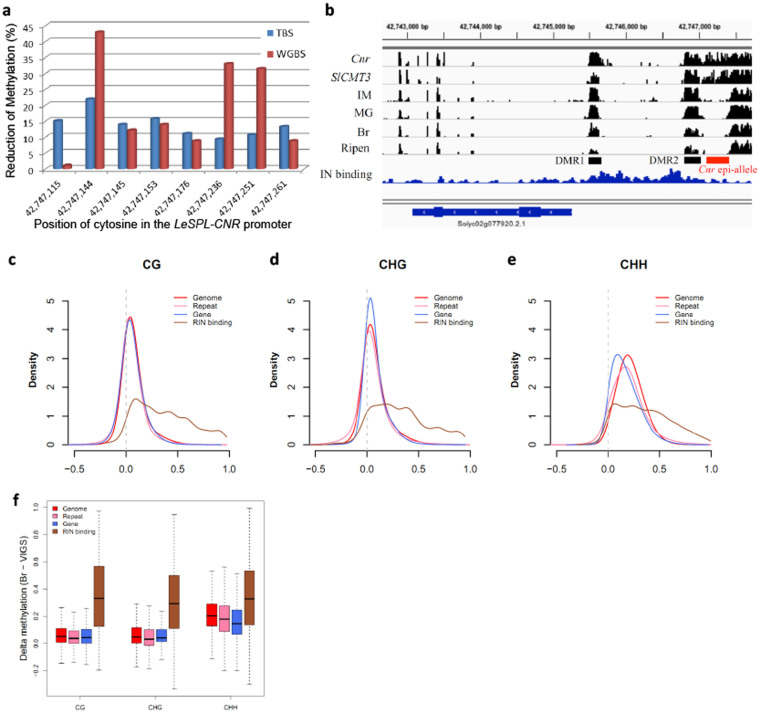
Analysis of single-base resolution methylome. (a–b), Targeted and whole-genome bisulfite sequencing (TBS, WGBS) reveals methylation changes in specific cytosine residues (a) and the overall *Cnr* promoter region (b) in the *SlCMT*-silenced *Cnr* fruit. Bar-chart shows the methylation levels in the *Cnr* gene locus in epimutant fruit at breaker stage (*Cnr*), *SlCMT3*-silenced *Cnr* fruit at breaker stage (VIGS), and in wild-type fruit at immature (IM), mature green (MG), breaker (Br), ripening stages (Ripen), and LeMADS-RIN ChIP-Seq (RIN binding). The location of the two differentially methylated regions (DMR1 and DMR2) and the epi-allele in the promoter region of *Cnr* are shown. (c–d), Genome-wide hypomethylation caused by *SlCMT3* silencing. Kernel density plots of the loss of CG (c), CHG (d) and CHH (e) methylation in the *SlCMT3*-silenced *Cnr* fruit at breaker stage. Methylation differences (methylation level of *Cnr* minus *SlCMT3* silenced *Cnr* fruit at breaker stage) of the whole-genome (bin = 1000 bp), annotated gene regions, repeats and the LeMADS-RIN bindings sites are shown, and regions with zero methylation are discarded^29^. (f), *SlCMT3* silencing causes global demethylation in *Cnr* fruit. Boxplot showing the delta-methylation levels of *Cnr* and *SlCMT3*-silenced fruits at the breaker stage. For calculation of the global methylation delta, genome is divided into 200-bp bins and the methylation levels of each bin are calculated. Gene and the repeat are defined according to the ITAG v2.5 annotation. RIN binding sites are called as previously described^29^.

Targeted-bisulfite sequencing^3^ was used to examine methylation in the 286-bp region, and its flanking sequences, of the *LeSPL-CNR* promoter in the *SlCMT3*-silenced epi-allele fruits. A marked reduction of methylation was observed at eight specific cytosines, seven at the CHG sites and one in the CG context among the eighteen cytosine residues that are fully methylated in *Cnr* (Fig. 4a; Supplementary Fig. 9a–i). No clear difference in methylation was observed up- and downstream of the 286-bp region. These results indicate that the hypermethylation status of the eight cytosines is critical for inhibition of the *LeSPL-CNR* promoter activity, and the reduction in methylation of these residues may allow an increase in *LeSPL-CNR* expression; resulting in the “*Cnr-to-ripening*” reversion in the epimutant fruits. Taken into account of the gene-specific VIGS (Fig. 1, Supplementary Table 1, Supplementary Figs. 3, 5, 6), the effect of *SlCMT3* reduction on the eight specific cytosine residues seems to refine the *Cnr* epi-allele in terms of functional hypotethylation.

### Effect of SlCMT3 silencing on whole-genome DNA methylation

The single-base resolution methylome of the *SlCMT3*-silenced *Cnr* fruit was further profiled by whole-genome bisulfite sequencing (WGBS), and confirmed the loss of methylation at the eight specific cytosines in the 286-bp promoter region (Fig. 4a, b). Moreover we observed that genome-wide hypomethylation occurred at CHG as well as CG and CHH sites in repeats and gene regions (Fig. 4c–e). It is unlikely that the occurrence of hypomethylation at CG and CHH sites was due to non-specific silencing of other *DMT* genes by PVX/SlCMT3-mediated VIGS (Fig. 1, Supplementary Table 1, Supplementary Figs. 3, 5, 6, 10), although the underlying mechanism for such reduction of methylation requires further investigation. On the other hand, it has been well-documented that LeMADS-RIN is required for the activation of fruit ripening genes by directly binding to promoters of those genes^21^,^28^,^29^. It has also been shown that LeMADS-RIN binding sites are demethylated in normal fruit and that LeMADS-RIN is unable to bind to the same sites in *Cnr* fruit due to a higher methylation level at those binding sites in *Cnr* than normal fruit^29^. We thus examined the methylation levels of LeMADS-RIN binding sites in our WGBS data and found that these sites became hypomethylated after *SlCMT3* silencing (Fig. 4f). These findings suggest that *SlCMT3* loss-of-function not only disrupted the *Cnr* epi-allele but might have also helped to elevate *LeMADS-RIN* expression (Fig. 3e, Supplementary Fig. 7c) that would allow functional restoration of the LeMADS-RIN activity for binding to these demethylated sites.

## Discussion

We describe a mechanism that maintains the stability of a naturally occurring epimutation, and thus of its associated phenotype in tomato. This mechanism relies on *SlCMT3*, possibly along with other key components such as *SlDRM7*, *SlCMT2* and *SlMET1*, in the RdDM and methylation maintenance pathways^6^,^7^,^8^,^9^,^10^,^11^,^12^,^13^,^14^. Silencing of *SlCMT3* in the epimutant fruits reduces methylation of eight specific cytosines mostly in the CHG context in the region of the *LeSPL-CNR* promoter and causes genome-wide hypomethylation, resulting in an up-regulation of *LeSPL-CNR* and key ripening genes and “*Cnr*-to-ripening” reversion.

**Figure 5:**
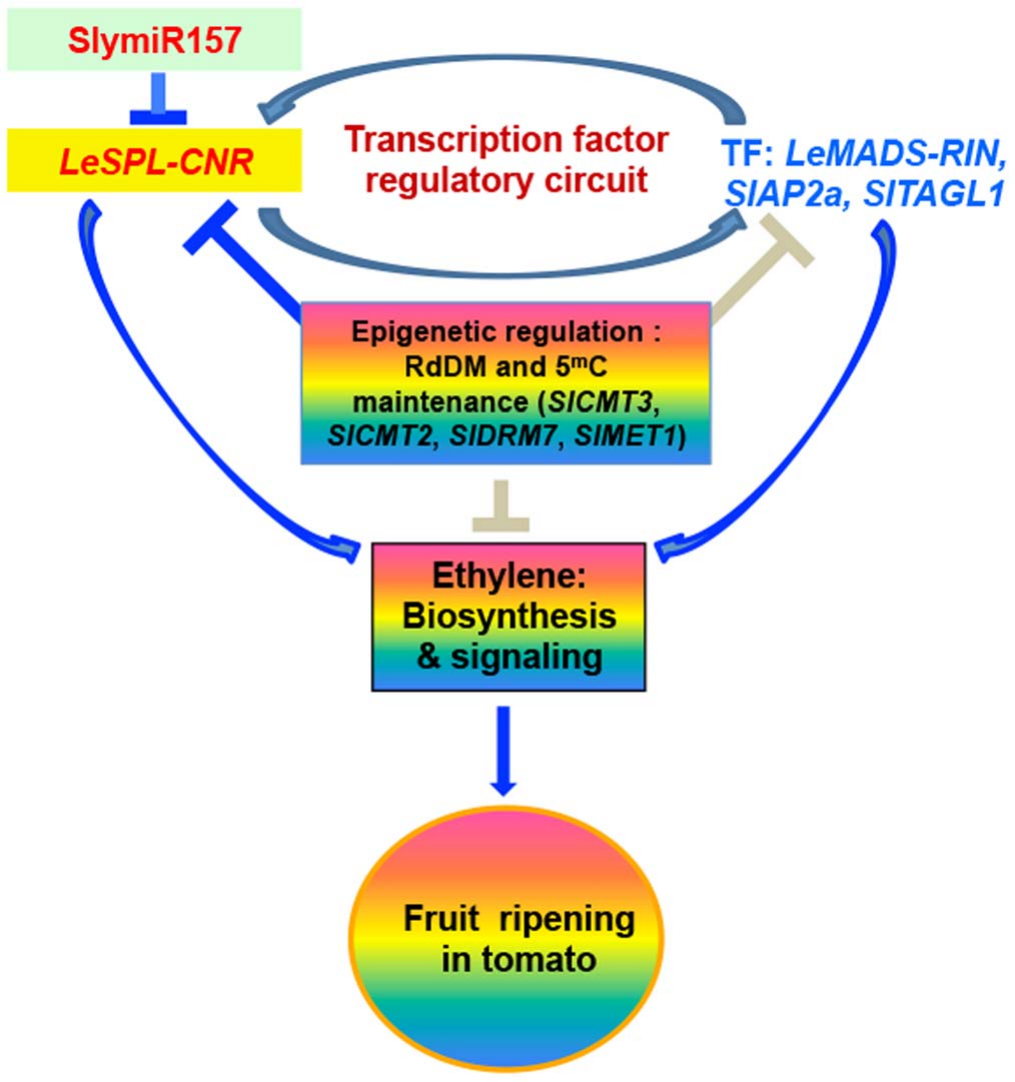
Maintenance of epigenetic stability in regulating tomato fruit ripening. *LeSPL-CNR* and key ripening-associated transcription factor (TF) genes form a regulatory circuit in the genetic and epigenetic control of tomato fruit ripening via modulation of ethylene biosynthesis and signal transduction. Regulation of *LeSPL-CNR* expression by SlymiRNA157 is also incorporated into this model. Blue arrow indicates activation while the “**T**” sign represents inhibition. Grey arrow and “**T**” sign indicate potential functional mode.

It is possible that the epi-allele *LeSPL-CNR* and key ripening-associated transcription factor (TF) genes including *LeMADS-RIN*, *SlAP2a* and *SlTAGL1* form a regulatory network that controls tomato development and fruit ripening. These TFs can regulate each other and they are involved in possible feedback loops in the genetic regulation of ripening^25^,^26^,^27^,^28^. TFs also regulate fruit ripening via transcriptional regulation of ethylene biosynthesis and signalling^25^,^26^,^27^,^28^. In tomato, DNA methylation may also contribute to fruit ripening^3^,^23^,^29^. Consistent with this hypothesis, the content of globally methylated cytosine (5^m^C) is under dynamic changes during tomato development and fruit ripening, and chemical-mediated demethylation can facilitate early premature ripening^24^,^28^,^31^,^32^,^33^. In *Cnr*, DNA methylation maintenance is critical for maintaining epigenetic stability of the naturally occurring epimutation. Silencing of key *SlDMTs* in RdDM and 5^m^C maintenance pathways can destabilise epigenetic status which is required to down-regulate *LeSPL-CNR*. Such negative epigenetic control may also play a direct or indirect role in modulation of key ripening-associated TFs, and ethylene biosynthetic and signalling genes. Furthermore, microRNAs may be also involved in the fine-tuning of *LeSPL-CNR* expression in modulation of tomato fruit ripening^34^. Taken together, this model suggests that TFs, ethylene structural and signal transduction genes, microRNAs, epigenetic maintenance and developmentally regulated epigenetic modifying genes such as *SlCMT3* involve tomato development and fruit ripening (Fig. 5).

In summary our results demonstrate that somatic maintenance of methylation may represent an essential layer of epigenetic regulation in addition to the complex genetic network for the stability of the *Cnr* epimutation and non-ripening phenotype. This idea is supported by that fruit development and ripening are associated with dynamic modifications of the whole-genome level of DNA methylation in normal tomato. Thus spontaneous, but stable, epigenetic mutations maintained by mechanisms such as those described in this work afford a new route for the evolution of modern plant species and in the case of crops such as tomato these altered phenotypes, if ‘beneficial', will be favored by natural selection and/or plant breeding.

## Methods

### Constructs

Non-translatable 300–525-bp fragments corresponding to the 5′ ends of each gene were PCR-amplified and cloned into the *Mlu*I/*Sal*I sites of the *Potato virus X* (PVX) vector^28^ to generate PVX/SlDRM7, PVX/SlMET1, PVX/SlCMT2, PVX/SlCMT3, and PVX/SlCMT4 (Fig. 1b). The 3′ UTR of the *SlCMT3* was also cloned into PVX to produce PVX/SlCMT3_UTR_. The full-length cDNA sequences of the nine tomato *DMT* genes and the sequences of the short non-translatable fragments that were used for construction of the PVX-based VIGS constructs are included in Supplementary Figure 10. A non-translatable *LeSPL-CNR* gene and the 286-bp region of the *LeSPL-CNR* promoter were cloned into the PVX/GFP vector^3^ to generate PVX/mLeSPL-CNR:GFP and PVX/Pcnr-GFP (Supplementary Fig. 1a). PVX encodes a RNA-dependent RNA polymerase (166 K), movement proteins (25 K, 12 K and 8 K) and capsid protein (CP). Primers are listed in Supplementary Table 2. All constructs were confirmed by sequencing.

### PVX-based gene silencing and plant growth conditions

PVX-based VIGS and Virus-induced transcriptional gene silencing in *Cnr*, *rin* and wild-type tomato (*Solanum lycopersicum* cv. Ailsa Craig) fruits were performed as described^3^,^22^. The carpopodium of tomato fruits at 5–15 days post anthesis was needle-injected with recombinant viral RNAs for each of the PVX-based VIGS constructs. Plants were grown in insect-free glasshouses at 25°C with supplementary lighting to give a 16-h photoperiod, examined and photographed with a Nikon Coolpix 995 digital camera.

### Quantitative real-time *PCR (qRT-PCR)*

Total RNA was extracted from tomato tissues using RNeasy Plant Mini Kit (Qiagen). cDNA was synthesized using a FastQuant RT Kit (Tiangen). qRT-PCR was performed on a Bio-Rad CFX96 Real-Time system (Bio-Rad) using an UltraSYBR Mixture Kit (CoWin Bioscience). At least three technical replicates for each of three biological replicates for each sample were analyzed. The relative level of specific gene expression was calculated using the formula 2^−ΔΔCt^ and normalized to the amount of 18S rRNA detected in the same sample as described^30^.

### Bisulfite sequencing

Total DNA was isolated from tomato tissues using DNeasy Plant Mini Kit (Qiagen). Bisulfite conversion, PCR amplification and sequencing were performed using the EZ DNA Gold Methylation Kit (Zymo Research), Blue MegaMix Double PCR mixture (Microzone) and BigDye Terminator Reaction Mixture (Applied Biosystems) as described^3^. Whole genome bisulfite sequencing and bioinformatics analysis were performed as previously described^29^.

## Author Contributions

W.C., J.K., C.Q. and Y.H. designed and performed experiments; J.T., C.W., H.W., Y.S., C.L., B.L., P.Z., Y.W., T.L. Z.Y., X.Z. and N.S. performed experiments; Y.C., S.Y. and S.Z. performed the WGBS and analysed the data; H.-z.W., T.O., Y.L., K.M., S.J., D.R., S.Z. and G.B.S. were involved in discussions and helped writing the paper; P.G. analysed data and helped writing the paper; Y.H. initiated the project, analysed data and wrote the paper.

## Supplementary Material

Supplementary Information Supplementary Information

## References

[b1] Patterson G. I., Thorpe C. J. & Chandler V. L. Paramutation, an allelic interaction, is associated with a stable and heritable reduction of transcription of the maize b regulatory gene. Genetics 135, 881–894 (1993). 7507455 10.1093/genetics/135.3.881 PMC1205727

[b2] Cubas P., Vincent C. & Coen E. An epigenetic mutation responsible for natural variation in floral symmetry. Nature 401, 157–161 (1999). 10490023 10.1038/43657

[b3] Manning K. *et al.* A naturally occurring epigenetic mutation in a gene encoding an SBP-box transcription factor inhibits tomato fruit ripening. Nature Genet. 38, 948–952 (2006). 16832354 10.1038/ng1841

[b4] Miura K. *et al.* A metastable DWARF1 epigenetic mutant affecting plant stature in rice. Proc. Natl. Acad. Sci. USA 106, 11218–11223 (2009). 19541604 10.1073/pnas.0901942106 PMC2708680

[b5] Silveira A. B. *et al.* Extensive natural epigenetic variation at a *de novo* originated gene. PLoS Genet. 9, e1003437 (2013). 23593031 10.1371/journal.pgen.1003437 PMC3623765

[b6] Lindroth A. *et al.* Requirement of CHROMOMETHYLASE3 for maintenance of CpXpG methylation. Science 292, 2077–2080 (2001). 11349138 10.1126/science.1059745

[b7] Jackson J., Lindroth A., Cao X. & Jacobsen S. Control of CpNpG DNA methylation by the KRYPTONITE histone H3 methyltransferase. Nature 416, 556–560 (2002). 11898023 10.1038/nature731

[b8] Zilberman D., Cao X. & Jacobsen S. ARGONAUTE4 control of locus-specific siRNA accumulation and DNA and histone methylation. Science 299, 716–719 (2003). 12522258 10.1126/science.1079695

[b9] Chandler V. & Stam M. Chromatin conversations: Mechanisms and implications of paramutation. Nature Rev. Genet. 5, 532–544 (2004). 15211355 10.1038/nrg1378

[b10] Chan S., Henderson I. & Jacobsen S. Gardening the genome: DNA methylation in Arabidopsis thaliana. Nature Rev. Genet. 6, 351–360 (2005). 15861207 10.1038/nrg1601

[b11] Alleman M. *et al.* An RNA-dependent RNA polymerase is required for paramutation in maize. Nature 442, 295–298 (2006). 16855589 10.1038/nature04884

[b12] Richards E. J. Inherited epigenetic variation - revisiting soft inheritance. Nature Rev. Genet. 7, 395–401 (2006). 16534512 10.1038/nrg1834

[b13] Henderson I. R. & Jacobsen S. E. Epigenetic inheritance in plants. Nature 447, 418–424 (2007). 17522675 10.1038/nature05917

[b14] Hirsch S., Baumberger R. & Grossniklaus U. Epigenetic variation, inheritance, and selection in plant populations. Cold Spring Harb. Symp, Quant. Biol. 77, 97–104 (2013). 23619013 10.1101/sqb.2013.77.014605

[b15] Law J. A. & Jacobsen S. E. Establishing, maintaining and modifying DNA methylation patterns in plant s and animals. Nature Rev. Genet. 11, 204–220 (2010). 20142834 10.1038/nrg2719 PMC3034103

[b16] Zemach A. *et al.* The Arabidopsis nucleosome remodeler DDM1 allows DNA methyltransferases to access H1-containing heterochromatin. Cell 153, 193–205 (2013). 23540698 10.1016/j.cell.2013.02.033 PMC4035305

[b17] Martin A. *et al.* A transposon-induced epigenetic change leads to sex determination in melon. Nature 461, 1135–1138 (2009). 19847267 10.1038/nature08498

[b18] Fujimoto R. *et al.* Epigenetic variation in the FWA gene within the genus Arabidopsis. Plant J. 66, 831–843 (2011). 21457364 10.1111/j.1365-313X.2011.04549.x

[b19] Durand S. *et al.* Rapid establishment of genetic incompatibility through natural epigenetic variation. Curr. Biol. 22, 326–331 (2012). 22285031 10.1016/j.cub.2011.12.054

[b20] Tarutani Y. *et al.* Trans-acting small RNA determines dominance relationships in Brassica self-incompatibility. Nature 466, 983–986 (2010). 20725042 10.1038/nature09308

[b21] Vrebalov J. *et al.* A MADS-box gene necessary for fruit ripening at the tomato ripening-inhibitor (*rin*) locus. Science 296, 343–346 (2002). 11951045 10.1126/science.1068181

[b22] Lin Z. F. *et al.* A tomato HD-Zip homeobox protein, LeHB-1, plays an important role in floral organogenesis and ripening. Plant J. 55, 301–310 (2008). 18397374 10.1111/j.1365-313X.2008.03505.x PMC2607530

[b23] Kanazawa A. *et al.* Virus-mediated efficient induction of epigenetic modifications of endogenous genes with phenotypic changes in plants. Plant J. 65, 156–168 (2011). 21175898 10.1111/j.1365-313X.2010.04401.x

[b24] Teyssier E. *et al.* Tissue dependent variations of DNA methulation and endoreduplication levels during tomato fruit development and ripening. Planta 228, 391–399 (2008). 18488247 10.1007/s00425-008-0743-z

[b25] Klee H. J. & Giovannoni J. J. Genetics and control of tomato fruit ripening and quality attributes. Annu. Rev. Genet. 45, 41–59 (2011). 22060040 10.1146/annurev-genet-110410-132507

[b26] Karlova R. *et al.* Transcriptome and metabolite profiling show that APETALA2a is a major regulator of tomato fruit ripening. Plant Cell 23, 923–941 (2011). 21398570 10.1105/tpc.110.081273 PMC3082273

[b27] Vrebalov J. *et al.* Fleshy fruit expansion and ripening are regulated by the tomato SHATTERPROOF gene TAGL1. Plant Cell 21, 3041–3062 (2009). 19880793 10.1105/tpc.109.066936 PMC2782289

[b28] Zhou T. *et al.* Virus-induced gene complementation reveals a transcription factor network in modulation of tomato fruit ripening. Sci. Rep. 2, 836 (2012). 23150786 10.1038/srep00836 PMC3495281

[b29] Zhong S. *et al.* Single-base resolution methylomes of tomato fruit development reveal epigenome modifications associated with ripening. Nature Biotechnol. 31, 154–159 (2013). 23354102 10.1038/nbt.2462

[b30] Qin C. *et al.* Involvement of RDR6 in short-range intercellular RNA silencing in *Nicotiana benthamiana*. Sci. Rep. 2, 467 (2012). 22737403 10.1038/srep00467 PMC3381291

[b31] Zhang M., Kimatu J. N., Xu K. & Liu B. DNA cytosine methylation in plant development. J. Genet. Genomics 37, 1–12 (2010). 20171573 10.1016/S1673-8527(09)60020-5

[b32] The Tomato Genome Consortium. The tomato genome sequence provides insights into fleshy fruit evolution. Nature 485, 635–641 (2012). 22660326 10.1038/nature11119 PMC3378239

[b33] Messeguer R., Ganal M. W., Steffens J. C. & Tanksley S. D. Characterization of the level, target sites and inheritance of cytosine methylation in tomato nuclear DNA. Plant Mol. Biol. 16, 753–770 (1991). 1859863 10.1007/BF00015069

[b34] Chen W. *et al.* Tuning *LeSPL-CNR* expression by SlymiR157 affects tomato fruit ripening. Sci. Rep. 5, 7852 (2015). 25597857 10.1038/srep07852 PMC4297963

