# Supplementary material for: Requirement of CHROMOMETHYLASE3 for somatic inheritance of the spontaneous tomato epimutation Colourless non-ripening
Source: Sci Rep. 2015 Mar 17;5:9192. doi: 10.1038/srep09192 (PMC4361866; doi:10.1038/srep09192)
Supplement: Supplementary Information [file srep09192-s1.pdf]

Requirement of *CHROMOMETHYLASE3* for somatic inheritance of the spontaneous tomato epimutation *Colourless non-ripening*

Weiwei Chen[1*], Junhua Kong[1*], Cheng Qin[1*], Sheng Yu[2*], Jinjuan Tan[1], Yun-ru Chen[2], Chaoqun Wu[1], Hui Wang[1], Yan Shi[3], Chunyang Li[3], Bin Li[1], Pengcheng Zhang[1], Ying Wang[1], Tongfei Lai[1], Zhiming Yu[1], Xian Zhang[1], Nongnong Shi[1], Huizhong Wang[1], Toba Osman[3], Yule Liu[4], Kenneth Manning[3], Stephen Jackson[3], Dominique Rolin[5], Silin Zhong[2], Graham B. Seymour[6], Philippe Gallusci[5], Yiguo Hong[1,3]

[1]Research Centre for Plant RNA Signalling, College of Life and Environmental Sciences, Hangzhou Normal University, Hangzhou 310036, China. [2]State Key Laboratory of Agrobiotechnology, School of Life Sciences, The Chinese University of Hong Kong, Hong Kong. [3]School of Life Sciences, University of Warwick, Coventry CV4 7AL, UK. [4]MOE Key Laboratory of Bioinformatics, Centre for Plant Biology, School of Life Sciences, Tsinghua University, Beijing 100084, China. [5]UMR Fruit Biology and Pathology, Bordeaux University, INRA, Villenave d'Ornon 33883, France. [6]Plant and Crop Science Division, University of Nottingham, School of Biosciences, Loughborough, Leics LE12 5RD UK.

Running title: *SlCMT3* modulates maintenance of tomato *Cnr* epiallele

[*]These authors contributed equally to this work.

Correspondence author: Yiguo Hong (yiguo.hong@warwick.ac.uk and yiguo.hong@hznu.edu.cn). Research Centre for Plant RNA Signalling, College of Life and Environmental Sciences, Hangzhou Normal University, Hangzhou 310036, China.

Co-correspondence author: Silin Zhong (silin.zhong@cuhk.edu.hk)

Supplementary Figure Legends

Supplementary Figure 1 | Post-transcriptional and transcriptional silencing of *LeSPL-CNR* inhibits fruit ripening. a, Outline of the *LeSPL-CNR* epilocus[3]. The two exons for the *LeSPL-CNR* mRNA and the hypermethylated ($5^{m}C$) region upstream of exon 1 are indicated. The 286-bp DNA fragment was PCR amplified and cloned into the *ClaI*/*Eag*I sites of PVX/GFP[3] to generate the ViTGS vector PVX/Pcnr-GFP. **b,** A wild-type AC fruit inoculated with PVX/GFP ripens normally. **c,** Post-transcriptional VIGS of *LeSPL-CNR* by PVX/mLeSPL-CNR blocks AC fruit to ripen, showing non-ripening sectors. PVX/mLeSPL-CNR expresses a non-translatable *LeSPL-CNR* mRNA that triggers silencing of endogenous *LeSPL-CNR* mRNA. **d-e,** Transcriptional ViTGS of *LeSPL-CNR* by PVX/Pcnr-GFP that contains specific RNA targeting the 286-bp region of *LeSPL-CNR* promoter inhibits fruit ripening. AC fruits injected with PVX/Pcnr-GFP RNA developed non-ripening sectors. **f,** Semi qRT-PCR assays with 23 and 28 cycles of amplification revealed that the level of *LeSPL-CNR* mRNA were down-regulated in non-ripening green (Gr) sectors when compared to that of the ripe red (Red) sectors of two treated AC fruits. Total RNAs extracted from the dissected green and red sectors were pre-treated with DNase I prior to being reversely transcribed into cDNA with (+) and without (-, as negative control) reverse-transcriptase (RT). **g-h,** Bisulfite sequencing shows induction of DNA methylation in the 286-bp *LeSPL-CNR* promoter region in non-ripening (filled rectangles, **h**), but not in ripe (open rectangles, **g**) sectors of AC fruits inoculated with PVX/Pcnr-GFP. Electrophoretogram shows

sequences corresponding to the 5' end (-2480 to -2403) of the 286-bp *LeSPL-CNR* promoter region[3](Supplementary Fig. 9i).

Supplementary Figure 2 | Phylogenetic relationship among tomato and Arabidopsis DNA methyltransferases (DMTs). Amino acid sequences of 10 AtDMTs including AtDRM1 (NM_121542.2, AT5G15380), AtDRM2 (NM_121466.2, AT5G14620), AtDRM3 (AY050882.1, AT3G17310), AtMET1 (NM_124293.3, AT5G49160), AtMET2 (NM_001203796.1, At4G14140), AtCMT1 (NM_106722.2, AT1G80740), AtCMT2 (NM_118020.4, At4G19020), AtCMT3 (NM_105645.3, AT1G69770), AT4G08990 (NM_116968.1) and AT4G13610 (NM_117435.1) were downloaded from the NCBI database. Amino acid sequences of 9 tomato putative DMTs including SlMET1 (AJ002140.1, SGN-U323958), SlCMT2 (SGN-U582753), SlCMT3 (SGN-U579786), SlCMT4 (SGN-U601004), SlDRM5 (NM_001246974.1), SlDRM6 (SGN-U575586), SlDRM7 (TC161581), SlDRM8 (SGN-U565936), and SlDRM9 (EST583972), were identified in the tomato EST databases (NCBI database, TIGR database, Solanaceae Genome Network). The protein sequences for SlCMT2, SlCMT3, SlCMT4, SlDRM7 and SlDRM9 were deduced from each of the corresponding full-length genes after RACEs (Supplementary Fig. 10). Phylogenetic relationship of DMTs was analyzed using ClustalW.

Supplementary Figure 3 | Silencing of *SlDMTs* by different VIGS vectors displays various impacts on ripening of *Cnr* epimutant fruits. a-d, *Cnr* fruits treated with

PVX/SlCMT2. **e-l,** *Cnr* fruits treated with PVX/SlDRM7. **m-n,** *Cnr* fruits treated with PVX/SlMET1. **o,** *Cnr* fruits treated with PVX/SlCMT4. **p,** Genetic *rin* mutant fruits treated with PVX/SlCMT3. Tomato fruits at 5-15 days post anthesis (dpa) were treated with PVX/SlCMT2, PVX/SlDRM7 or PVX/SlMET1 and these fruits broke and subsequently ripened, showing sectors with different degrees of ripening as well as non-ripening sectors (**a-n**). Ripening was assessed by red colour and pericarp softening as observed in the ripe wild-type AC fruits (Supplementary Fig. 5h). No ripening was observed in *Cnr* fruits inoculated with PVX/SlCMT4 (**o**). Genetic *rin* mutant fruits at 5-15 dpa were treated with PVX/SlCMT3 and these fruits remained unripe, showing characteristic of the *rin* phenotype (**p**). Photographs were taken at the indicated day post-anthesis.

Supplementary Figure 4 | Silencing of *SlCMT3* triggers early breaker of *Cnr* fruits. *Cnr* fruits at 5-15 days post anthesis (dpa) that were mock-inoculated (Mock), or inoculated with the empty VIGS vector PVX, PVX/SlDRM7 (DRM7), PVX/SlMET1 (MET1), PVX/SlCMT2 (CMT2), PVX/SlCMT3 (CMT3), PVX/SlCMT3$_{UTR}$ (CMT3$_{UTR}$) or PVX/SlCMT4 (CMT4) broke at 38.6±2.2 (n=90), 37.2±2.5 (n=90), 38.1±1.6 (n=30), 37.7±2.5 (n=60), 35.9±1.3 (n=30), 34.1±2.6 (n=60), 36.0±2.4 (n=30), 38.7 ±1.6 (n=30) dpa, respectively. These data suggest that silencing of *SlCMT3* influenced tomato fruit ripening. Breaker was judged when fruits started to turn from mature green to red or yellow. Asterisk (*) indicates statistical significance between the *SlCMT3*-silenced and the PVX control samples by Student's *t*-tests ($p<0.001$).

Supplementary Figure 5 | Silencing of *SlCMT3* by PVX/SlCMT3 reverses *Cnr* epimutant fruits to complete ripening. a-f, *Cnr* fruits were treated with PVX/SlCMT3 at 5-15 days post anthesis (dpa) and these fruits broke and subsequently ripened fully. Ripening was assessed by red colour and pericarp softening as observed in the ripe wild-type AC fruits (**h**). Ripening *Cnr* fruits show sectors of red-ripening (RR), weak ripening (WR, light-red) and yellow-ripening (YR, mixed yellow-red), as indicated in (**e**). RR, WR and YR sectors eventually ripened completely. **g,** No ripening was observed in *Cnr* fruits inoculated with PVX. **h,** Ripening of wild-type AC fruits. Photographs were taken at the indicated day post-anthesis.

Supplementary Figure 6 | Silencing of *SlCMT3* by PVX/SlCMT3$_{UTR}$ causes *Cnr* epimutant fruits to ripening. a-i, *Cnr* fruits at 5-15 days post anthesis (dpa) were treated with PVX/SlCMT3$_{UTR}$ and these fruits broke and subsequently ripened, showing sectors of red-ripening (RR), weak ripening (WR) and yellow-ripening (YR). Ripening was assessed by red colour and pericarp softening as observed in the ripe wild-type AC fruits (Supplementary Fig. 5h). Photographs were taken at the indicated day post-anthesis.

Supplementary Figure 7 | *SlCMT3* silencing affects expression of *LeSPL-CNR* and key ripening TF genes. a-f, Relative levels of endogenous *SlCMT3* (**a**), *LeSPL-CNR* (**b**), *LeMADS-RIN* (**c**), *LeHB1* (**d**), *SlAP2a* (**e**), and *SlTAGL1* (**f**) mRNAs in red-ripening

(RR), weak-ripening (WR) and yellow-ripening (YR) sectors of *Cnr* fruits inoculated with PVX/SlCMT3$_{UTR}$ (SlCMT3) at 41 days post inoculation. Student's *t*-tests indicates a statistical significance (p<0.001) of increase of the RNA level of each gene tested in the *SlCMT3*-silenced samples when compared to that in the PVX control (asterisked). Level of mRNA of each gene in the mock-inoculated *Cnr* fruits (Mo) was included as references. **g-h,** Developmental regulation of master ripening TF gene expression. Semi-qRT-PCR reveals levels of mRNA of each TF gene in wild-type AC (**g**) and epimutant *Cnr* (**h**) young leaves (YL), fully opened flowers (OF) and pericarp tissues from fruits at immature green (IMG), mature green (MG), breaker (B), breaker + three days (B+3) and breaker + nine days (B+7) stages. The number of PCR cycles is indicated at the right of each panel. These results together with data in Fig. 3 suggest that in addition to maintaining the methylation of repetitive DNA and transposon-related sequences[31], *CMT3* may also have a novel role in modulation of regulatory genes.

Supplementary Figure 8 | Silencing of *SlCMT3* affects expression of ethylene synthetic and signalling transduction genes. a-f, Relative levels of endogenous *SlACS1* (**a**), *SlACS2* (**b**), *SlACS4* (**c**), *SlACO1* (**d**), *SlEBF1* (**e**) and *SlEBF2* (**f**) mRNAs in red-ripening (RR), weak-ripening (WR) and yellow-ripening (YR) sectors of *Cnr* fruits inoculated with PVX/SlCMT3$_{UTR}$ (SlCMT3) at 41 days post inoculation. Student's *t*-tests indicates a statistical significance in increase (p<0.001) of the RNA level of each gene tested in the *SlCMT3*-silenced samples when compared to that in the

PVX control (asterisked). Level of mRNA of each gene in the mock-inoculated *Cnr* fruits (Mo) was included as references.

Supplementary Figure 9 | Silencing of *SlCMT3* reduces cytosine methylation in the epimutated region of the *LeSPL-CNR* promoter. a-h, Targeted bisulfite sequencing shows the eighteen fully methylated cytosines in the 286-bp region of the *LeSPL-CNR* promoter in *Cnr* (solid-rectangles, **g-h**), but not in wild-type AC fruits (open-rectangles, **a**). *SlCMT3* silencing reduced cytosine methylation in the CHG (red-asterisked) and CG (green-asterisked) context in red-ripening (RR), weak red-ripening (WR) and yellow-ripening (YR) sectors of fruits injected with PVX/SlCMT3 (Cnr+SlCMT3, **d-f**) or PVX/$SlCMT3_{UTR}$ (Cnr+$SlCMT3_{UTR}$, **b-c**). The increasing height of the thymidine (T) peak indicates a shift from methylated to non-methylated cytosine (asterisked). **i,** Nucleotide-coordinates correspond to the numbering of the *LeSPL-CNR* sequence[3]. Numberings of the eight hypomethylated cytosines in the *Cnr* promoter region of *SlCMT3*-silenced fruits are also indicated in terms of their positions in the tomato genome sequence[32].

Supplementary Figure 10 | Full-length cDNA sequences of nine tomato *DMT* genes. **a,** *SlMET1*. **b,** *SlCMT2*. **c,** *SlCMT3*. **d,** *SlCMT4*. **e,** *SlDRM5*. **f,** *SlDRM6*. **g,** *SlDRM7*. **h,** *SlDRM8*. **i,** *SlDRM9*. Sequences highlighted red or blue indicate relevant sequences of corresponding cDNA fragments that were cloned into the PVX-based VIGS vector (Fig. 1b) for gene-specific RNA silencing of each of the targeted *DMT* genes.

Supplementary Table 1 Nucleotide sequence similarities among VIGS targets and their full-length tomato DMT genes

	SlMET1^{FL}	***SlMET1***^{VIGS}	***SlCMT2***^{FL}	***SlCMT2***^{VIGS}	***SlCMT3***^{FL}	***SlCMT3***^{VIGS}	***SlCMT3-UTR***^{VIGS}	***SlCMT4***^{FL}	***SlCMT4***^{VIGS}	***SlDRM5***^{FL}	***SlDRM6***^{FL}	***SlDRM7***^{FL}	***SlDRM7***^{VIGS}	***SlDRM8***^{FL}	***SlDRM9***^{FL}
SlMET1^{FL}	100.0%														
SlMET1^{VIGS}	100.0%	100.0%													
SlCMT2^{FL}	28.4%	n.h	100.0%												
SlCMT2^{VIGS}	28.3%	n.h	100.0%	100.0%											
SlCMT3^{FL}	28.1%	n.h	30.2%	34.5%	100.0%										
SlCMT3^{VIGS}	34.7%	n.h	n.h	n.h	100.0%	100.0%									
SlCMT3-UTR^{VIGS}	29.0%	n.h	n.h	n.h	100.0%	44.9%	100.0%								
SlCMT4^{FL}	27.7%	n.h	27.2%	31.3%	28.3%	40.5%	n.h	100.0%							
SlCMT4^{VIGS}	26.9%	n.h	30.5%	30.5%	31.4%	n.h	n.h	100.0%	100.0%						
SlDRM5^{FL}	28.6%	n.h	28.4%	31.3%	30.0%	32.2%	34.9%	29.1%	30.9%	100.0%					
SlDRM6^{FL}	27.7%	n.h	28.7%	32.3%	28.5%	29.5%	25.0%	29.4%	30.9%	78.0%	100.0%				
SlDRM7^{FL}	26.8%	n.h	28.2%	29.1%	30.1%	25.9%	32.2%	28.8%	30.5%	75.3%	75.4%	100.0%			
SlDRM7^{VIGS}	30.4%	n.h	29.6%	29.6%	28.3%	n.h	n.h	30.4%	30.6%	29.6%	32.6%	100.0%	100.0%		
SlDRM8^{FL}	30.0%	n.h	27.0%	29.3%	27.8%	25.0%	40.9%	28.0%	29.1%	40.4%	40.4%	40.9%	33.0%	100.0%	
SlDRM9^{FL}	28.0%	n.h	28.5%	32.3%	28.7%	28.1%	30.2%	29.1%	30.9%	77.2%	99.6%	75.6%	32.6%	40.1%	100.0%

FL: Nucleotide sequences of full-length genes were used for comparisons. VIGS: VIGS-targeting sequences, which were cloned into the PVX vector for silencing specific genes (Fig. 1b), were used for comparisons. n.h: no nucleotide sequence homology was found.

Supplementary Table 2 Primers used for this study

Primer/Construct	Sequences (5' - 3')	Gene or fragment amplified	Annealing temperature	Detection/Cloning sites
**Primers**				
**PP269**	CAAGGTGCGCGAGGTTTACCAATC	PVX mRNA (154bp)	50℃	qRT-PCR for PVX virus RNA
**PP269-R**	CCCCTCTAGATCATTAGCCGCTTC			accumulation
**PP271**	CGGCTACCACATCCAAGGAAGG	18S rRNA (188bp)	50-65℃	Control gene
**PP272**	GAGCTGGAATTACCGCGGCTG			
**SlCMT3-S**	AGTACCAGTTGCCCGGGCTTT	SlCMT3 (131bp)	50℃	qRT-PCR for *SlCMT3* expression
**SlCMT3-A**	GAGATTCGTCATTGGAGACCAGTTC			
**LeSPL-CNR-F**	TGGAAACTAACAAATGGGAAGGG	LeSPL-CNR (207bp)	58℃	PCR for *LeSPL-CNR* expression
**LeSPL-CNR-R**	GGCGATGGTATGGCTTGG			
**LeMADS-RIN-F**	AAGGAACCCAAACTTCATCAG	LeMADS-RIN (119bp)	58℃	PCR for *LeMADS-RIN* expression
**LeMADS-RIN-R**	TTGTCCCAAATCCTCACCTA			
**LeHB1-F**	CTACGACGAGCAGTCACCG	LeHB1 (161bp)	58℃	PCR for *LeHB1* expression
**LeHB1-R**	GGAACCATACAGCCACCT			
**SlAP2a-F**	GGAGTATGAATCCGATGAAGGT	SlAP2a (184bp)	58℃	PCR for *SlAP2a* expression
**SlAP2a-R**	CGATTCCAAATTGTGGTCTT			
**SlTAGL1-F**	GCAATAACTCCCTGCCTGTA	SlTAGL1 (140bp)	55℃	PCR for *SlTAGL1*expression
**SlTAGL1-R**	AGATGAAGAGCCTTGACCC			
**SlACO1-F**	CACTAACGGGAAGTACAAGAG	SlACO1 (101bp)	58℃	qRT-PCR for *SlACO1* expression
**SlACO1-R**	CTGCATCACTTCCTGGATTGTA			
**SlACS1-F**	GTGCTTCAAACAAAGGGAC	SlACS1 (146bp)	50℃	qRT-PCR for *SlACS1* expression
**SlACS1-R**	GTCCTAACCAAAGGCGAAT			
**SlACS2-F**	GTAGGTGTTGAGAAAAGTGGAG	SlACS2 (162bp)	55℃	qRT-PCR for *SlACS2* expression
**SlACS2-R**	GTCTTAACGAACTAATGGTGAGG			

**SlACS4-F**	ACACCGATTAACCGAGAGGC	SlACS4 (131bp)	50℃	qRT-PCR for *SlACS4* expression
**SlACS4-R**	CCCGTGGACGTAGCCAATTT			
**SlEBF1-F**	ATTGCCATCACTGACATAGC	SlEBF1 (111bp)	58℃	qRT-PCR for *SlEBF1* expression
**SlEBF1-R**	AGTTATAGCAAGCGACCTC			
**SlEBF2-F**	TTACCAGGTGTGTGGAAGG	SlEBF2 (136bp)	58℃	qRT-PCR for *SlEBF2* expression
**SlEBF2-R**	CCGACATTAGTAATACCACGA			
**SlMET1i-F**	CGACGACAAGACCCTCTGCTAAGCGGCACAACC	SlMET1 (250bp)	58℃	Construction of pRNAi-SlMET1
**SlMET1i-R**	GAGGAGAAGAGCCCTATTTGCCTGTGCTTGTGC			(intermediate vector, unpublished)
**SlCMT2i-F**	CGACGACAAGACCCTCCGATGCAGGATCCCAA	SlCMT2 (436bp)	58℃	Construction of pRNAi-SlCMT2
**SlCMT2i-R**	GAGGAGAAGAGCCCTAAGGGAGATCAGAAAGTG			(intermediate vector, unpublished)
**SlCMT3i-F**	CGACGACAAGACCCTCAAGGTACTAACCTCAAC	SlCMT3 (217bp)	58℃	Construction of pRNAi-SlCMT3-3 and
**SlCMT3i-R**	GAGGAGAAGAGCCCTGTCAATCCAATGTAGAAA			the silencing trigger RNA (intermediate
				vector, unpublished)
**SlCMT3i-F1**	CGACGACAAGACCCTGTTACACGAGCAGAGCC	SlCMT3 (275bp)	58℃	Construction of pRNAi-SlCMT3-5 and
**SlCMT3i-R1**	GAGGAGAAGAGCCCTGGTGGTAATGTAAGTAATGG			the silencing trigger RNA (intermediate
				vector, unpublished)
**SlCMT4i-F**	CGACGACAAGACCCTAAGGTGCGATGGA	SlCMT4 (253bp)	58℃	Construction of pRNAi-SlCMT4
**SlCMT4i-R**	GAGGAGAAGAGCCCTTTTCATCAGACAAT			(intermediate vector, unpublished)
**SlDRM7i-F**	CGACGACAAGACCCTGTTTACAATGAGAAGT	SlDRM7 (260bp)	58℃	Construction of pRNAi-SlDRM7
**SlDRM7i-R**	GAGGAGAAGAGCCCTTATGTTCGGGTAGTGGA			(intermediate vector, unpublished)
**Constructs**				
**PVX/SlMET1**	RNAi/PVX-F: GATACGCGTCCTTCGCAAGACCCTTCC	SlMET1 fragment (359bp)	58℃	*Mlu*I/*Sal*I
	RNAi/PVX-R:			
	TTCGTCGACTTACCAAGGGCCCTGAGGAG			
**PVX/SlCMT2**	RNAi/PVX-F: GATACGCGTCCTTCGCAAGACCCTTCC	SlCMT2 fragment (525bp)	58℃	*Mlu*I/*Sal*I

	RNAi/PVX-R: TTCGTCGACTTACCAAGGGCCCTGAGGAG			
**PVX/SlCMT3UTR**	RNAi/PVX-F: GATACGCGTCCTTCGCAAGACCCTTCC<br>RNAi/PVX-R: TTCGTCGACTTACCAAGGGCCCTGAGGAG	3' UTR portion of SlCMT3 mRNA (306bp)	58℃	*Mlu*I/*Sal*I
**PVX/SlCMT3**	RNAi/PVX-F: GATACGCGTCCTTCGCAAGACCCTTCC<br>RNAi/PVX-R: TTCGTCGACTTACCAAGGGCCCTGAGGAG	5' fragment of SlCMT3 mRNA (364bp)	58℃	*Mlu*I/*Sal*I
**PVX/SlCMT4**	RNAi/PVX-F: GATACGCGTCCTTCGCAAGACCCTTCC<br>RNAi/PVX-R: TTCGTCGACTTACCAAGGGCCCTGAGGAG	SlCMT4 fragment (342bp)	58℃	*Mlu*I/*Sal*I
**PVX/SlDRM7**	RNAi/PVX-F: GATACGCGTCCTTCGCAAGACCCTTCC<br>RNAi/PVX-R: TTCGTCGACTTACCAAGGGCCCTGAGGAG	SlDRM7 fragment (349bp)	58℃	*Mlu*I/*Sal*I

Supplementary Figure 1 (Hong)

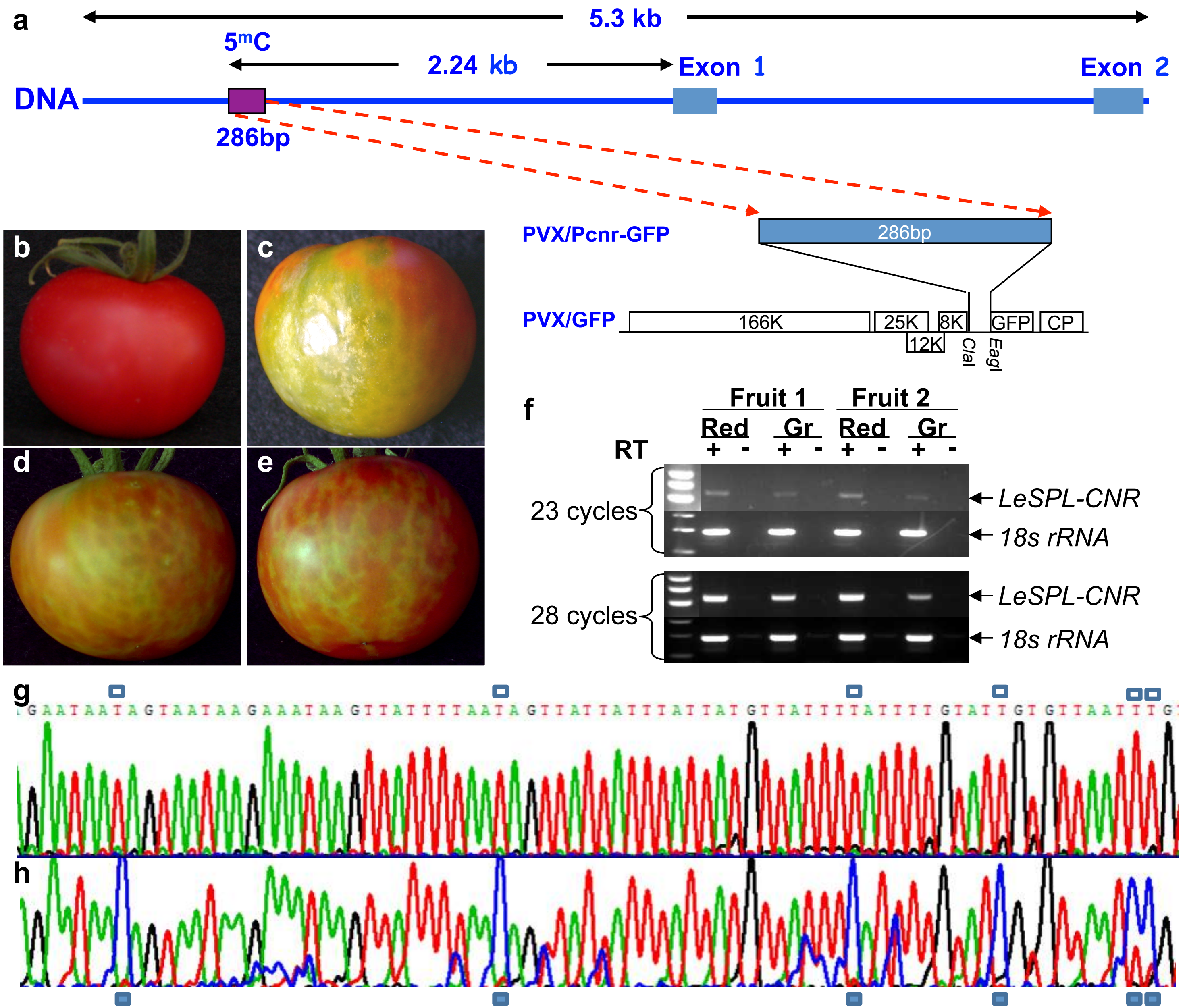

Supplementary Figure 2 (Hong)

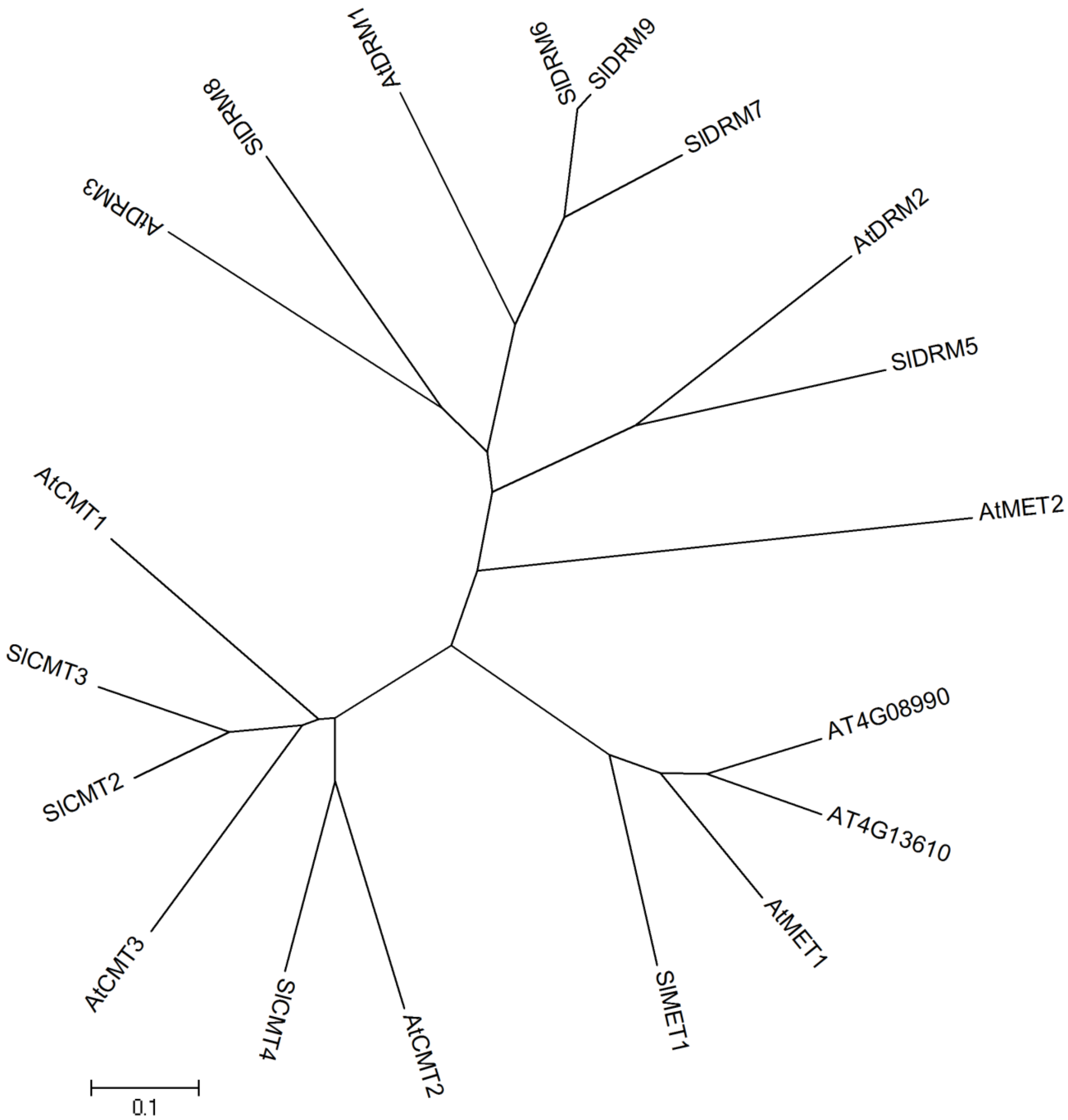

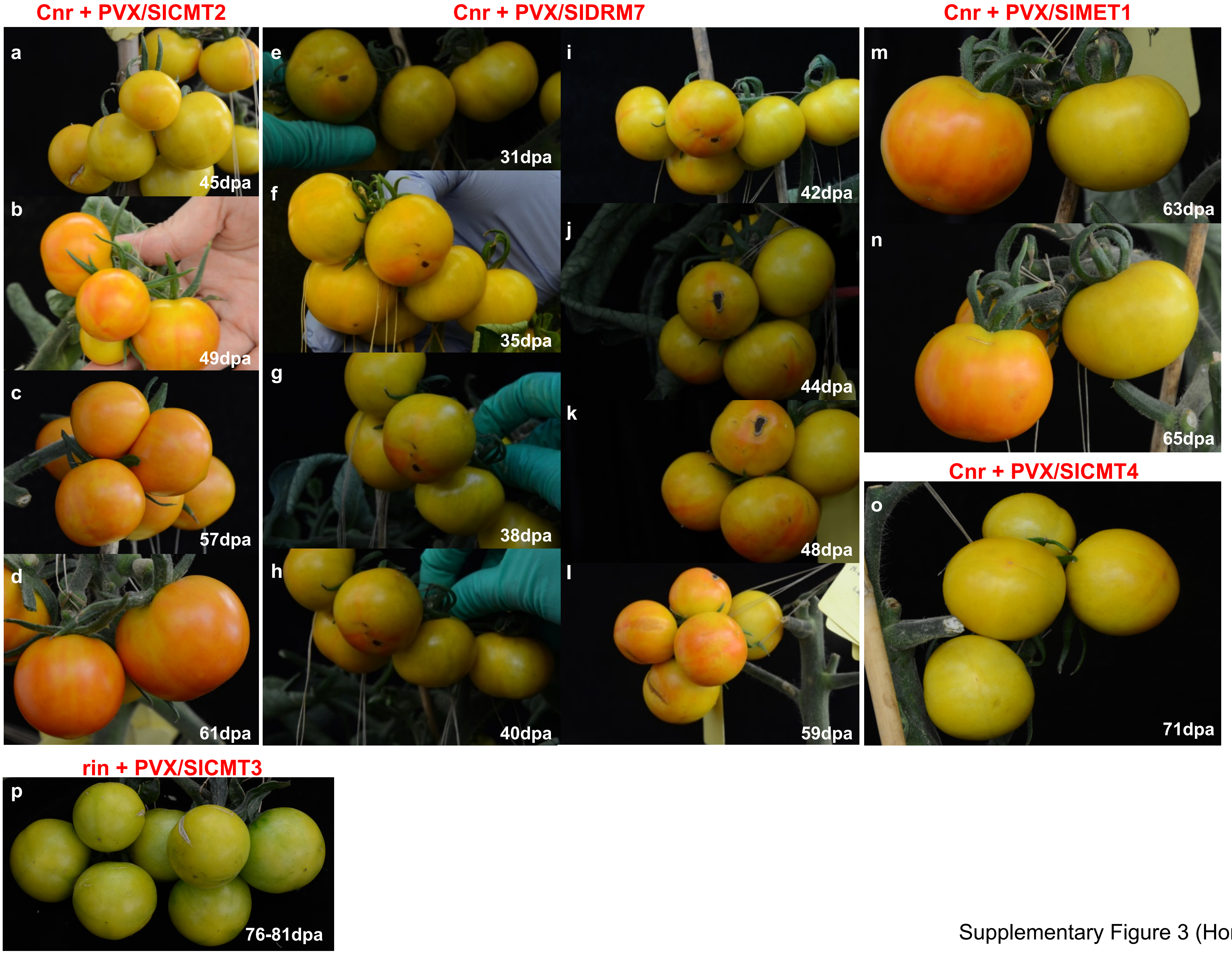

Supplementary Figure 3 (Hong)

Supplementary Figure 4 (Hong)

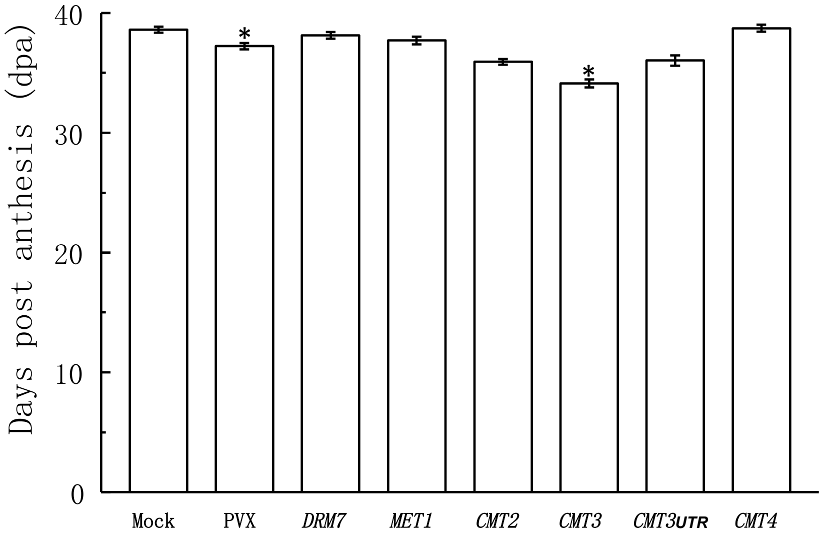

Supplementary Figure 5 (Hong)

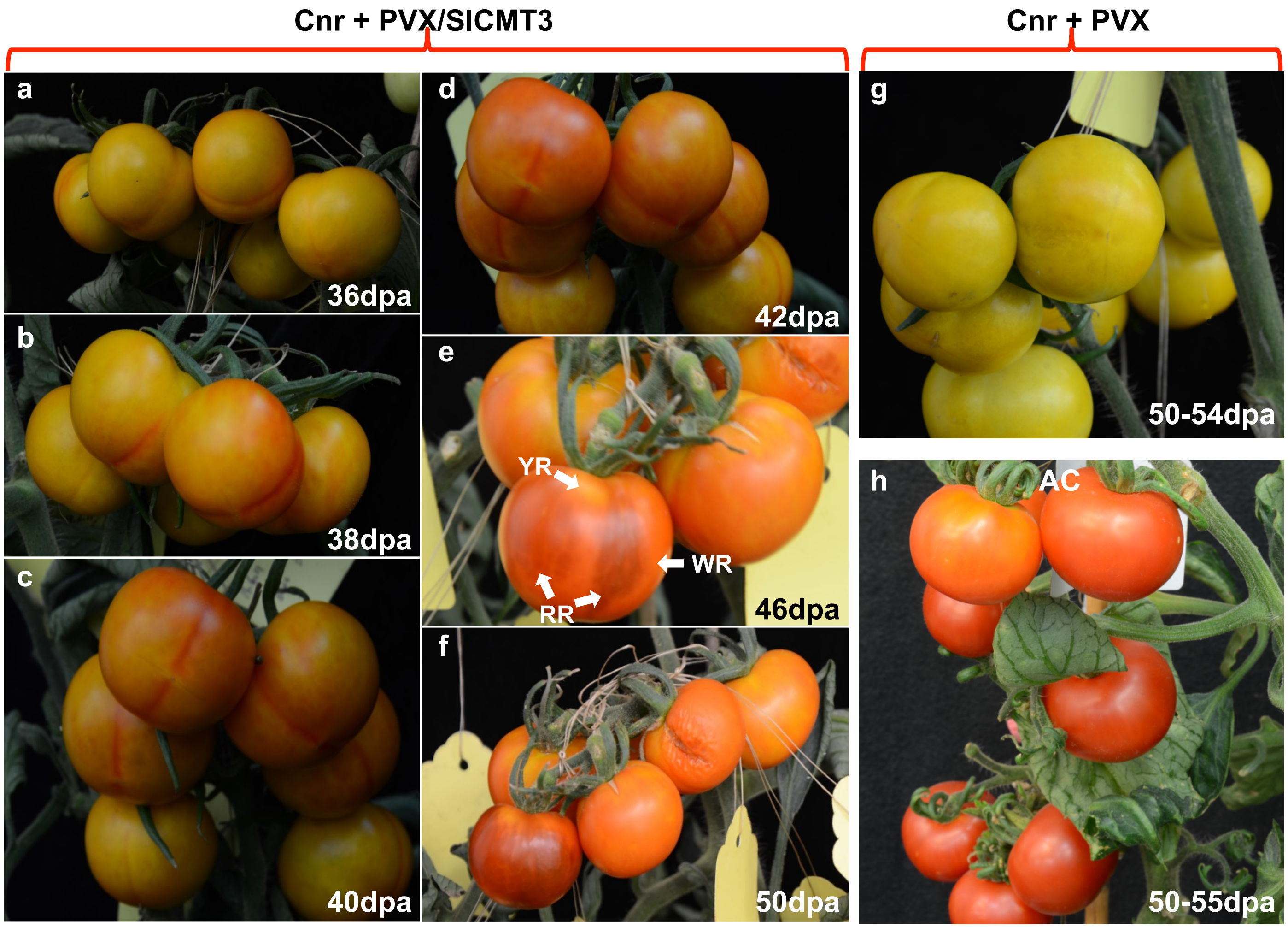

Supplementary Figure 6 (Hong)

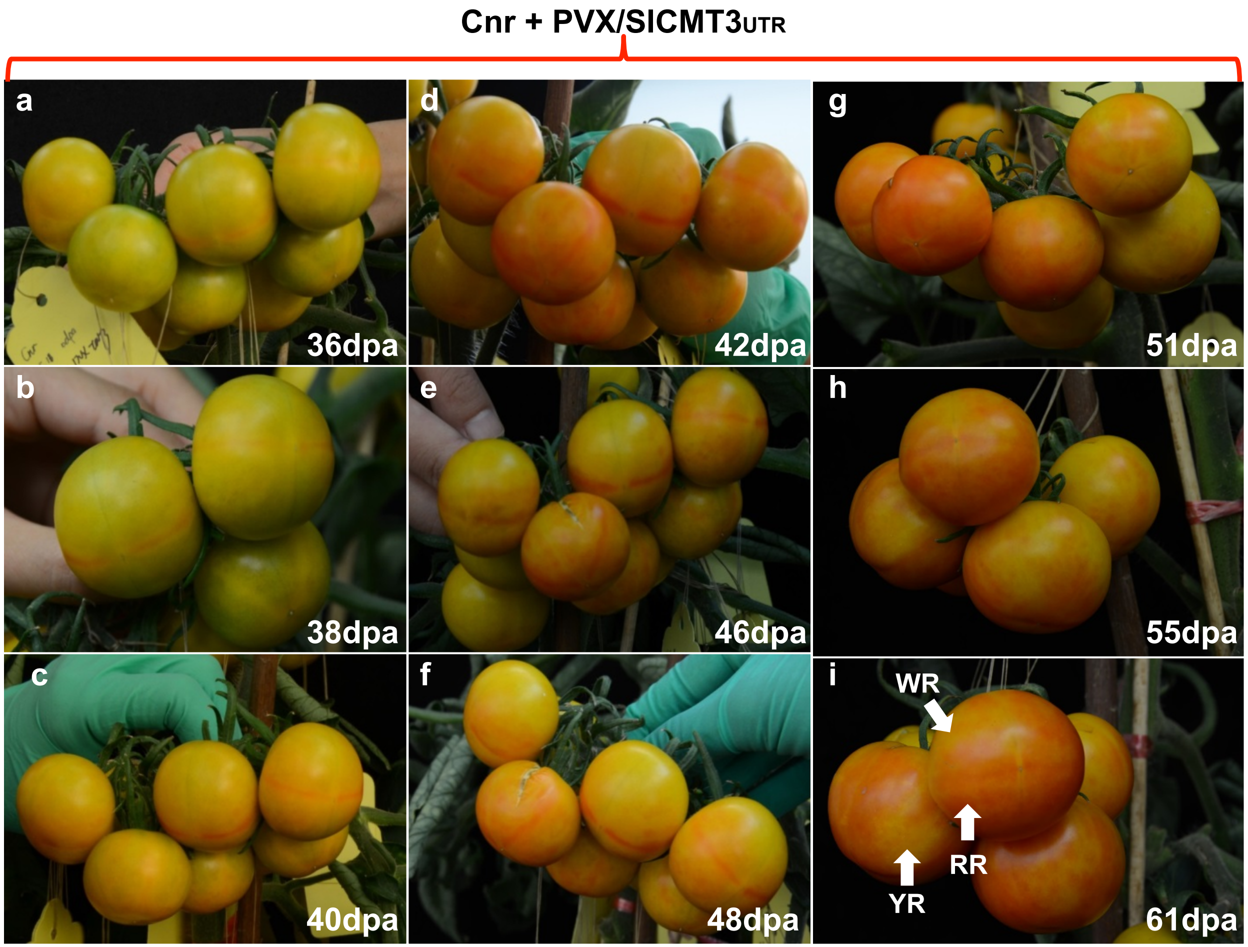

Supplementary Figure 7 (Hong)

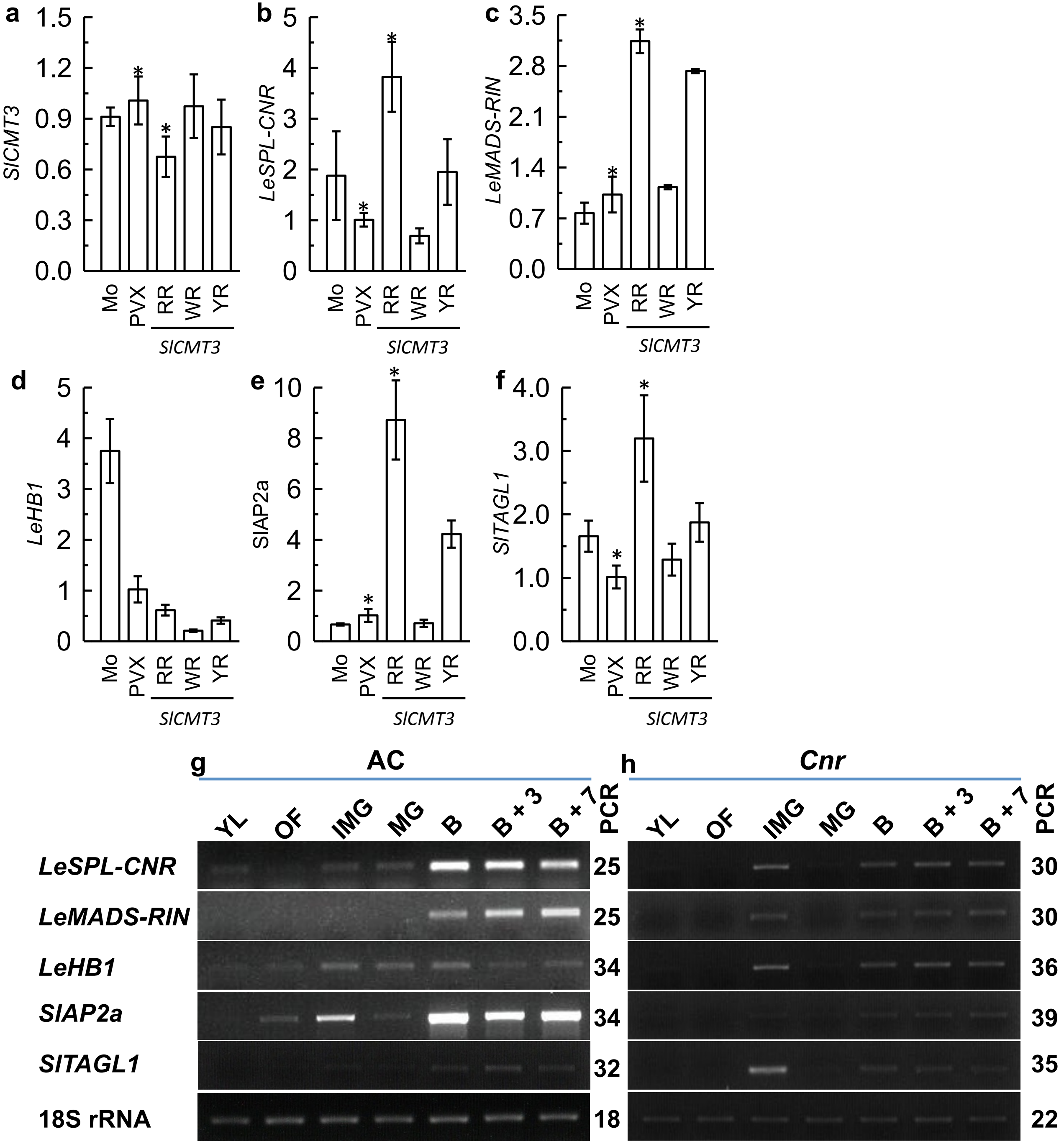

Supplementary Figure 8 (Hong)

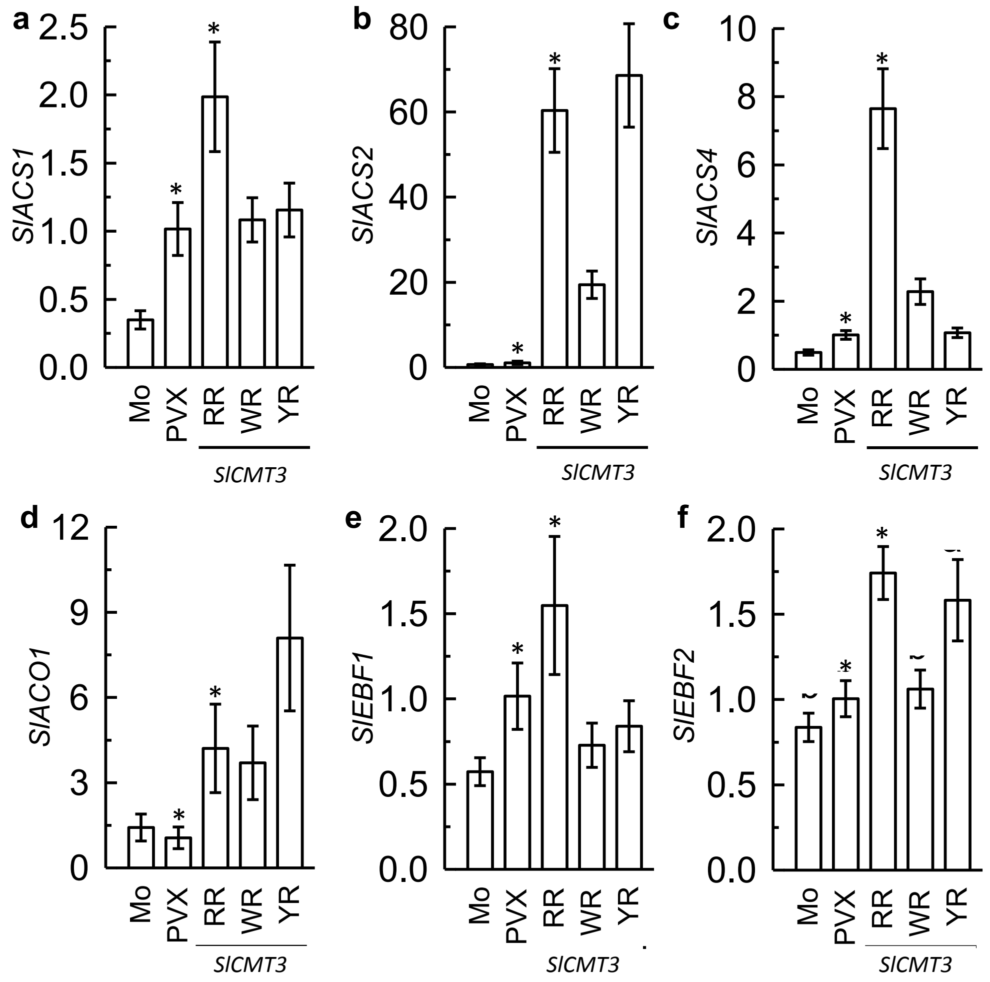

Supplementary Figure 9 (Hong)

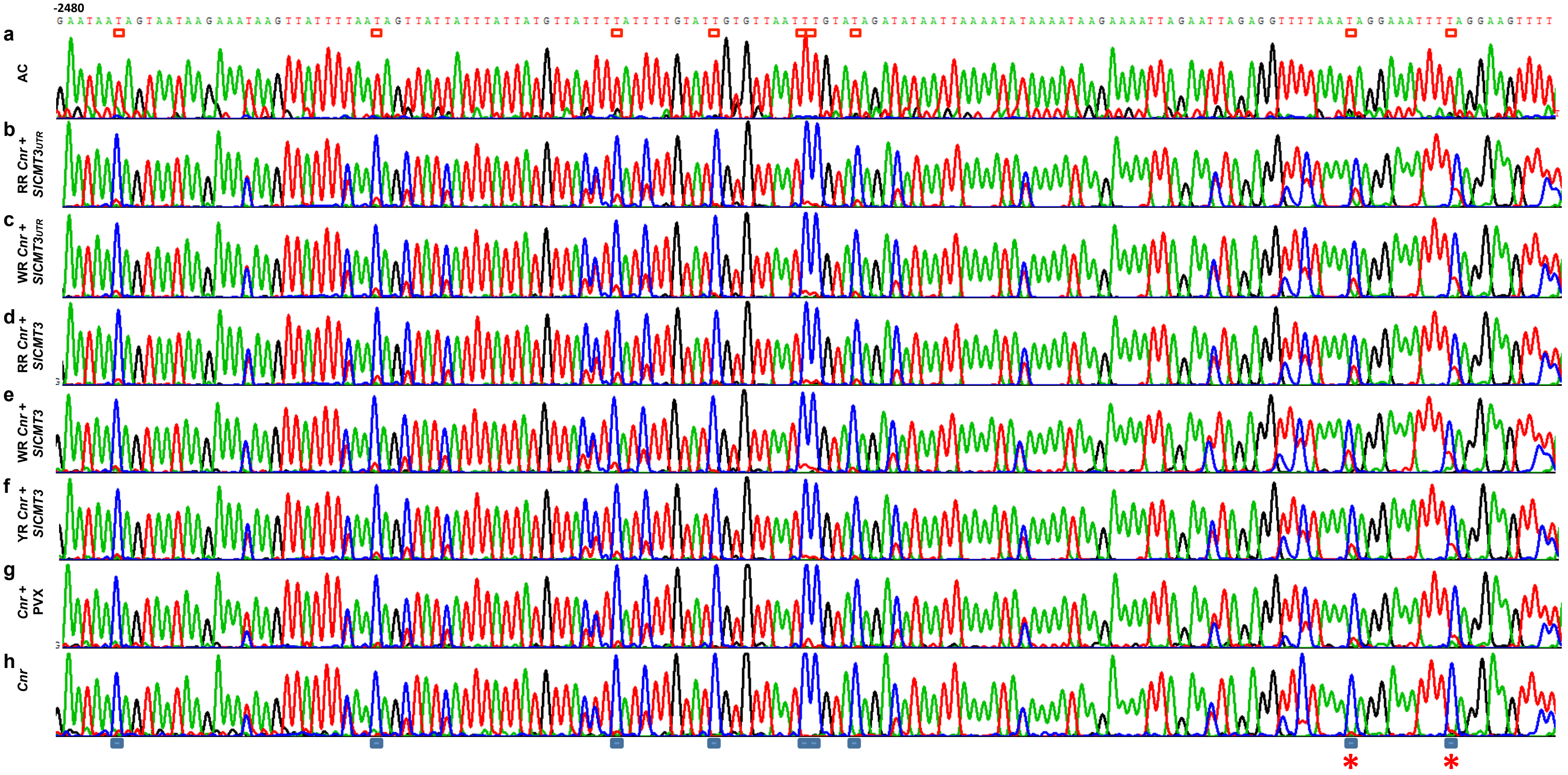

Supplementary Figure 9 Cont. (Hong)

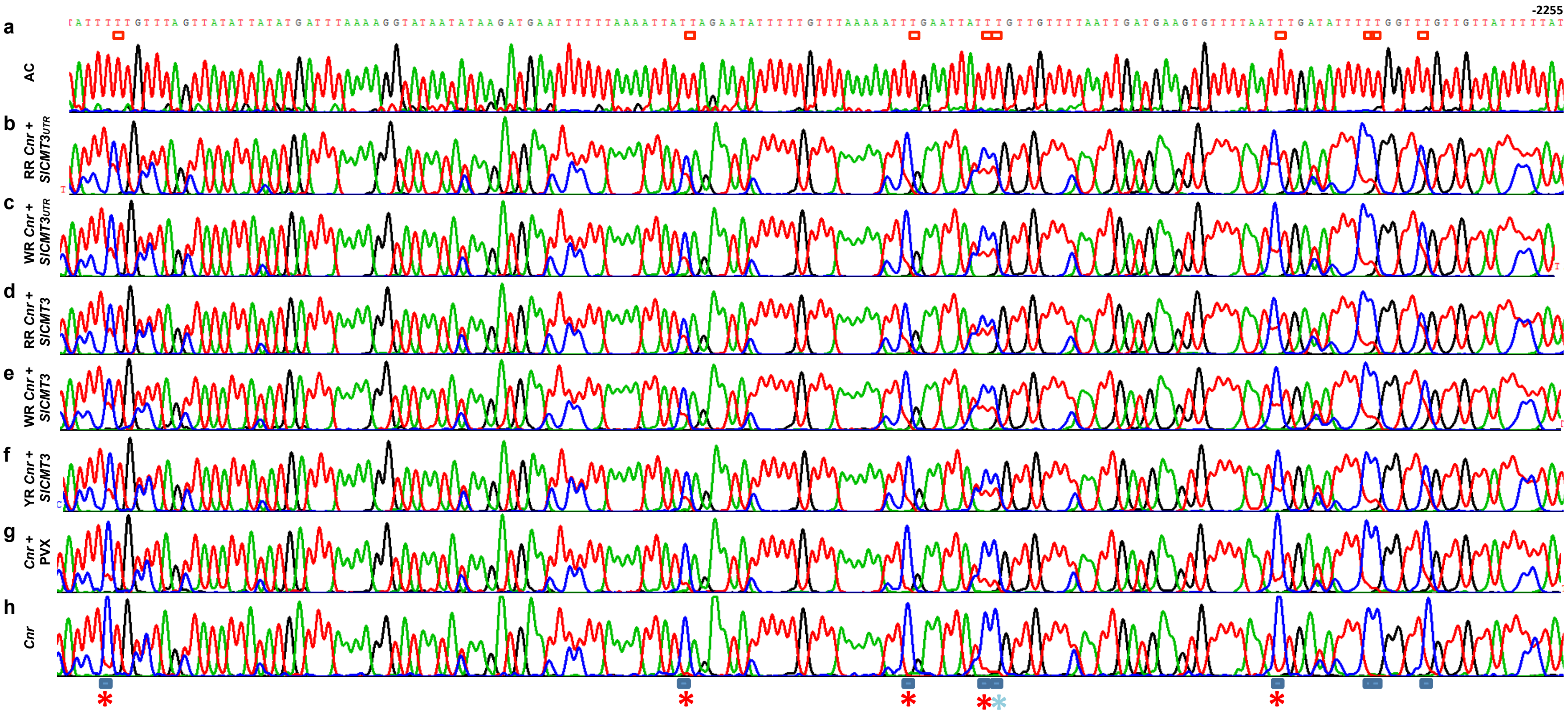

Supplementary Figure 9 Cont. (Hong)

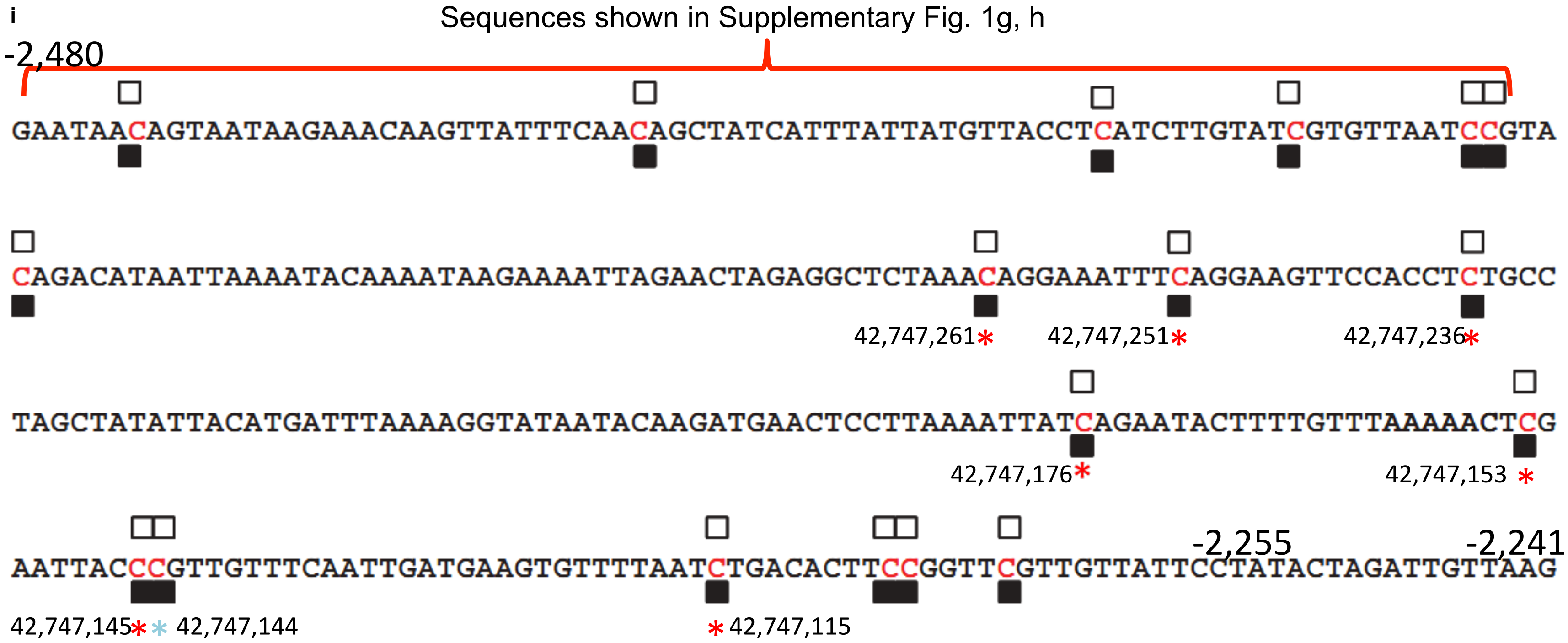

a

SlMET1 (Sequences highlighted RED was cloned into PVX/SlMET1)

CCCGCCCAAATCCCCCCAAAAACCTATCTCATTTGTCCTCTTCCTGTTGGAGAACTCAGCAACAGC
ACACCCCATCTCCCTCAACTTCTCCGCCGCACCAGCTTCTACTCTCCATTTCCGCCGAAAAATCAC
CTTTCACCGGCAAAGCAGCAGCCTCTGGTCCCTCCTTTATCTTCTCCCTTCGCTGCTCTGGCCACC
ATCGCTGGTCGCTGGCGAGAACTACAACGAAAATCCCTTCGCCTCCGCTCTCCTCTCTCCCTCTTC
CGCCGCCCTGCTCCTCACTTCTCACTTCTCCATTGAAGTCGACGGACGGGATAACGGCAGCGACGA
CTGCTCCGCCCAGCTACTGTGGCGAAGTAGCAGCAAGCTGTGACCAGCAAACTCGGCAAACTCCGG
CCAAAGCAGCGATAACAACTCAGGCCAGCAGTGGGGCAGCAACGACTGCTCTGCCCAGCAGCTTTG
GCGACGGCAAGCTGTGACCAGCAAACTCCGGCGAAGCAGCGATAACAACTCAGGCCAGCAGTGGGG
CAGCAACTCCGGTTGGTGAGGATGGCGTCACCCCAACCTAATTCGGAGTCGGTATTAGAACTTCCG
AACAACGACAAATCTGGACACAAAAAGAACAAACGCAAACAAGATTCTGTGTCAAAAAGGAAGGCA
TCTGCAACTGGTAAGAAGGAAAAGAAACAGGCTGTTTCTGAAACTATTGAGGAGCCCACTGCTGGA
CGTAAAAGGCCTAAGCGAGCTGCTGCCTGTTCAGATTTTAAAGAGAAATCTGTGCATTTTATCAAAA
AAGTCTTCAGTCATTGAAACAAAGAAGGACCATTGTGTAGACGAAGAGGATGTAGCTATTAGGTTA
ACTGCGGGTCTGCAAGAGTCTCAACGACCCTGTAGAAGATTAACGGATTTTGTTTTTCATAACTCA
GAAGGAATACCACAACCGTTTGGAATGTCTGAGGTTGATGATCTGTTTATCAGTGGCCTCATTTTA
CCACTTGAGGACAGTCTTGACAAAGTAAAAGCAAAAGGAATTAGATGTGAAGGCTTTGGGCGTATT
GAAGAATGGGCTATCTCTGGCTATGAAGATGGAACTCCTGTCATATGGATCTCAACTGAGACAGCT
GATTATGATTGTTTAAAACCCTCAGGTAGTTATAAGAAGTTTTATGACCACTTCTTGGCCAAGGCG
ACGGCTTGCGTTGAGGTTTATAAGAAGCTTTCAAAGTCATCTGGAGGGAATCCTGATTTAAGTCTT
GACGAGTTGCTTGCAGGGGTTGTCCGAGCGATGACTGGCATAAAATGCTTTTCAGGTGGAGTATCC
ATCAGGGACTTTGTCATCACTCAGGGCGGGTTCATATATAAGGAACTTATTGGTCTGGATGATACA
TCAAAGAAGACTGATCAACTTTTTGTTGAGCTACCTGTCCTAGCTTCCCTTAGAGATGAAAGCAGC
AAGCACGAGACACTTGCACAACCAGAGACTATATCATCTGGTAATGGTCTACGTATTGGCCCAAAA
GCAGGAAATGGAGGAGACAAGATAGTTGAATCTGGTTTGGCCAATGGTCCAGCGCCAGAAGATGAA
GATCTAAAATTGGCTAAATTGTTGCATGAAGAGGAGTATTGGTGCTCCTTGAAGCAGAAGAAAGAC
CGTAATACATCTTCCTCATCCAGCAAAATATACATCAAGATCAATGAGGATGAGATTGCAAGTGAT
TATCCTTTACCTGCATATTACAAAACATCTAATGAAGAGACTGATGAGTATATTGTCTTTGACAGT
GGGGTTGAAACATACCATATTGATGAGTTGCCTCGCAGCATGCTTCATAATTGGGCATTATACAAC
TCGGACTCAAGGCTAATATCTTTAGAACTGCTGCCAATGAAAGCTTGTGCTGATATTGATGTAACC
ATTTTTGGGTCTGGAGTGATGACTGCTGATGATGGGTCTGGCTACAATTTTGACACAGATGCTAAT
CATTCCTCTTCAGGTGGTTCTAGATCAGCTGAAATTGATGGAATGCCAATTTACCTGAGTGCTATA
AAAGAATGGATGATTGAGTTTGGGTCCTCAATGATCTTTATATCAATTCGGACTGATATGGCCTGG
TATAGGCTTGGGAAGCCATTGAAACAGTATGCTCCTTGGTACGAACCAGTCATAAAGACTGCAAGA
TTGGCAGTGAGCATCATTACTTTGTTAAAGGAACAGAATCGTGTGGCTAGACTTTCTTTTGGAGAA
GTTATTAAAAGGGTTTCAGAGTTCAAGAAAGACCATCCTGCTTATATATCATCTAATGTAGATGCA
GTGGAAAGGTATGTGGTTGTACATGGGCAAATTATTCTCCAGCAGTTTTCTGAATTTCCTGATGTA
AGCATTAGGAATTGTGCATTTGCGGTTGGTCTCTCAAGGAAAATGGAAGAGAGGCACCATACAAAA
TGGGTGATTAAGAAGAAGAAGGTGATGCAGAGACTGGAACAGAACTTAAATCCTAGAGCATCTATG
GCGCCATCTGTAAAAAGGAAAGCTATGCAGGCTACTACAACAAGGCTAATCAACAGAATCTGGGGG
GAATACTATTCCAATTACTCACCCGAGGTGTCAAAGGAGGTGGCTGATTGTGAGGTGAAGGATGAT
GAAGAACCAGATGAGCAAGAGGAAAATGAAGAGGATGATGTTCCGGAGAGGAACTTGGATGTTCCA
GAGAAAGCTCATACACCTTCTTCTACAAGAAGGCATATTAAGTCACGTTCTGACAGCAAAGAAATA

AACTGGGATGGGGAATCCATAGGTAAAACAGCTTCTGGTGAACAGTTGTTTAAAAAAGCTAGAGTT
CATGGACATGAGATAGCTGTTGGAGATTCAGTTCTAGTGGAACATGATGAACCAGATGAGCTTGGT
TGTATTTACTTTGTTGAATACATGTTTGAAAAATTGGATGGTAGCAAAATGCTTCATGGAAAAATG
ATGCAACGAGGATCTGACACTGTACTTGGAAATGCAGCTAATGAGAGAGAGGTATTTTTGATCAAT
GAATGCATGAATCTGCAACTAGGAGATGTCAAAGAAAGTATAGCTGTCAATATCAGAATGATGCCT
TGGGGACACCAGCATAGAAACACGAATGCTGATAAACTTGAAACAGCAAAAGCAGAAGACAGAAAG
AGGAAGGGATTGCCGACGGAATTTTACTGCAAAAGCTTTTATCGCCCTGAAAAAGGTGCTTTTTTC
AGACTCCCGTTTGATAAGATGGGCCTTGGTAATGGTTTATGCTACTCTTGTGAGTTGCAGCAAACT
GATCAGGAAAAGGAATCCTTTAAGTTTGATATGTCCAAATCCAGTTTTGTATATCTGGGGACTGAG
TATTCAGTTGATGACTTTGTTTATGTAAGCCCCGATCACTTTACTGCAGAAAGAGGGGGAAATGGA
ACTTTCAAAGCCGGAAGAAATGTGGGGTTGATGGCCTATGTAGTATGTCAATTACTAGAAATTGTT
GGACCTAAGGGATCTAAACAAGCTAAAGTAGATTCTACAAATGTTAAAGTCAGAAGATTCTTCAGA
CCAGAGGATATATCTTCAGATAAGGCATACCTCTTCTGATATCCGGGAGATCTATTACAGTGAAGA
TATACATACAGTTCCTGTGGAAATAATCAAAGGAAAATGTGAAGTGAGGAAGAAGTATGATATTTC
CTCTGAAGATGTCCCTGCCATGTTCGACCATATTTTCTTTTGTGAATATTTGTATGATCCATTGAA
TGGATCCCTTAAGAAGTTACCAGCTCAGATAAACCTGAGATTGTCAAAAATTAAGCTAGATGACGC
AACATCTAGGAAGAGGAAGGGGAAGGGAAAAGAAGGAGTGGATGAAGTTGGGGAACTAAATGAAAC
TTCTCCACAGAATCGTTTGTCCACATTAGATATCTTTGCTGGTTGTGGTGGCTTGTCTGAGGGGTT
GCAGCATTCGGGTGTCACAGATACAAATTGGGCAATTGAATACGAAGCGCCTGCTGGAGATGCATT
TAGACTTAATCATCCAAAGACAAAGGTGTTCATACATAATTGCAATGTGATTTTGAGGGCTGTCAT
GCAGAAGTGTGGAGATTCTGATGACTGTATCTCAACTCCAGAGGCTTCTGAATTAGCTGCAGCAAT
GGATGAGAGCGAACTGAATAGTTTGCCACTGCCTGGACAAGTTGATTTCATTAATGGAGGCCCTCC
TTGTCAGGGGTTTTCTGGAATGAATAGATTTAATCAGAGCACCTGGAGTAAAGTACAGTGTGAGAT
GATTCTGGCATTTTTATCCTTTGCTGATTATTATCGGCCCAAGTTTTTTCTCTTGGAGAATGTTAG
GAATTTTGTTTCGTTCAACCAAAAACAAACATTTCGCTTAACTGTTGCTTCCCTTCTTGAGATGGG
TTATCAGGTTAGGTTTGGTATCCTTGAAGCCGGAGCGTATGGAGTTCCTCAGTCTAGGAAGAGAGC
ATTTATCTGGGCTGCCTCCCCAGAGGAGGTTCTTCCAGAGTGGCCAGAACCAATGCATGTTTTTGC
TGTCCCAGAATTAAAAATCGCATTATCTGAAACTTCATACTATGCAGCTGTGAGGAGTACTGCTAG
TGGAGCTCCATTCCGTTCACTTACTGTCAGAGACACAATTGGAGATCTTCCTGTTGTTGGCAATGG
GGCAAGCAAGACTTGCATAGAGTATCAAGGTGATCCAGTATCCTGGTTCCAAAAGAAAATCCGGGG
CAGCTCAATAACATTATCTGATCACATTTCAAAAGAGATGAATGAGCTTAACCTAATCAGGTGCCA
AAGAATCCCCAAGCGGCCAGGAGCTGATTGGCGTGACCTTGAAGATGAAAAGGTTAAACTATCTAA
TGGTCAACTAGTTGATTTGATTCCATGGTGCCTGCCTAACA***CTGCTAAGCGGCACAACCAGTGGAA***
GGGGCTCTTTGGAAGGTTGGATTGGATGGGAACTTCCCCACTTCTATTACTGATCCCCAGCCGAT
GGGCAAGGTGGGGATGTGCTTTCATCCAGATCAAGACAGGATTGTTACAGTTCGTGAATGTGCACG
TTCTCAAGGTTTCCCAGACAGCTACCAATTTGCTGGTAACATCTTGCACAAGCACAGGCAAATAGG
AAATGCTGTTCCACCTCCTTTGGCATATGCGCTTGGAAGAAAACTCAAAGAAGCTGTTGAGAGCAA
AAATAGGCTCACTTAGAACTTTTTTAAGCTGTGAATTTTACATGCATGTCAATTACCATTCACATT
GCCAAATTATATCAGTTACTCATTTATTAAATTTGCAGTTTCACCTATAACCCTCTATTTAGAGGT
TGGGTTCAAACAAAAAAAATTAAAACAAAAAAAAA

b

SlCMT2 (Sequences highlighted RED was cloned into PVX/SlCMT2)

CAGAGAAAATCAGTGAGTTTGTGGTGAAAGGCTTCAAAGCAAATCTTTTGCCGTTGCCTGGTGATG

TTGATGTTGTATGCGGTGGACCACCTTGCCAAGGAATCAGTGGGTTCAATCGTTTTAGGAATAAAG
AAAAT***CCGATGCAGGATCCCAAAAATAAACAACTTGATGTCTACATGGACATTGTGGATTTCTTGA***
AACCCAGGTTTGTATTAATGGAGAATGTGGTGGACCTTGTCAAATTCTCCAATGGTTTCCTTGGGC
GATATGCATTGAGCAGACTTGTAGGGATGAACTACCAAGCACGGATGGGAATGATGGCAGCTGGTG
CGTATGGTCTTCCACAATTTCGTATGCGTGTTTTCATGTTTGGAGCTCTTTCATCAGAGAAGTTGC
CACAATATCCATTGCCCACACATAAAGTTATTGTGAGGGGTGTTATTCCCGTAGAATTTGAGTCAA
ACACAGTTGCGTATGATTCAGTCAGGGATCTCGAGTTAAAGAAAGAACTCTTTCTTGGTGATGCAC
TTTCTGATCTCCCTTTGGTGGAGAACAATGAGCCAAGAGATGAAATGCCTTACACTGATGAGCCTA
AATCTGATTTCCAGCATTTTATAAGAATGGGGAGGGATGGGTTGTTGGGAAGCGTACTATATGATC
ATCGTCCTCTTCAGTTGAACGAAGATGATCATCAGCGTGTATGTCAAATTCCAAAGCGGAAGGGTG
CAAACTTCAGGGACTTGCCCGGAGTTCGTGTTCGGCCAGATAATAAAGTTGAATGGGACCCAGACG
TGGAGAGAGTAAAGCTACCTTCTGGGAAGCCTTTGGTCCCTGACTATGCGATGAGCTTCGTTGGTG
GTAGTTCCTCAAAACCATTTGGTCGTTTGTGGTGGGATGAAACTGTCCCAACAGTTGTGACGAGAG
CTGAACCCCATAATCAGACCATAGTACATCCACTACAAGATAGAGTGCTCACAATTCGTGAAAATG
CAAGGCTCCAAGGTTTTCCTGACTACTACAAGTTGATAGGTCCAATAAAAGAAAGGTACATGCAAG
TAGGTAATGCAGTTGCAGTTCCAGTTGCCCGAGCATTGGGCTATTCTTTGGCCATGTCAATAAAGG
GATTATCTGGAGAAACACCATTGTTCACCTTGCCAAAAAATTTCCCTTCTCATGAGGATCAGAATT
GTAATGAAGTGTCACAATAATTTAAACATTAACTAATTGAGATTACCAAACTATCTTTCACCCTCT
GTATGGGGGATGATTATTTTTAGGATCTTAGTTTTATTGATTAATTTGCCTTCAGACATTGTTTCA
CAATACAATCATGCCAATGTTGAAGCTGGTGAAGGCGTGGGTTTATGTAATAGCAAGGTGAACATG
TGTCATGTTCTATTCTAGTTCCCAGAAAATGTATTTTGAGCATTCAATTCCATGTAACATTTCAAG
TTGTAAACTGTCAGAAAACATTTAGACACATTAAAAAAAAAAAAAAAAAAAAA

c

SlCMT3 (Sequences highlighted Red is cloned into PVX/SlCMT3; sequences highlighted Blue is cloned into PVX/SlCMT3UTR))

CGGAGAAATTGTGCTGGATAGTGAAGCTAGGAAAAAATGGCCTCATAGATATATTTTAAAGGATAA
CGTGAATATAAATAGTGCATCTATGAGCTTAAATTGCCAACATGATTCGGACGAATTAATTCAGGC
CAAGTGCCATTTTGCACAAGCATTAGTTGATAATGTCATTTATAAACTTGGGGATGATGCATATGT
AAAGGCTGCAGAAGATGAAGATGATTACATATGCAAAATTGTTGAATTTTTTCAAGGTGTTGATGA
TATGAAGTATTTTACTGCTCAGTGGTTTTACAGAGCGAAGGATACCGTAATTAAAGCTCATGACCA
GTTTATTGACAAGAAGCGTGTATTCTTGTCAGATATTAAGGATCACAACCCGCTTGATTGCCTCGT
AAAAAAAATCAAAATTGTTCCAATATCCTCAAACGTAAGCTTACAATTCAAGGAAAGTTTGCGATT
AGAATGTGACTACTACTATGACATGAAGTACCTTGTCCCATTCTCATCATTTATTAGTTTACCATC
AGATGTTTTAAGTCCTGATAGTGAATCAAATTCGACCATATCAAGTGACGGTGATGTTGTGGAGGT
CAAAGAACAGAAGCAAGAAAAGAAACTCTTGGATCTTTATTCCGGTTGTGGTGGAATGTCTACTGG
GCTGTGCCTGGGTGCTGATGTTTGTGATGTTAAACTTGTCACTAAATGGACTGTTGATCTAAATTG
TTATGCCTGTGATAGTTTAAAAGCAAACCACCCAGAAACCGAGGTGAGAAATGAATCTGCTGAAGA
TTTCTTATTGCTTTTGAAGGAGTGGGAGCAGCTTTGTGCATCCTGCTCCTTATTGAAAAGCAATAC
CCCGACACATCCTTTGTTGAAAGTGGGAGATGCAAATGTGGAAGATGATGATGAAGGTGCAGATGA
TGATGATGGGGGATCTGGTGATGAAGATGAAGGTGAAATTTTTGAAGTGGAAGAGATCTTGGAAGT
TTGTTATGGAGACCCGAAGGAAATAAAAAAGCCAGGCCTTTATTTTAAGGTACGATGGAAGGGTTA
TGGCCCAGATGAAGATACTTGGGAGCCTATAGAAGGCTTAGATGGCTGCCAAAACAAAATAAAAGA
CTTTGTGACTGATGGCTTTAAAAGAAGTGTTTTGCCATTACCTGGGCAAGTAGACGTCGTATGTGG

GGGACCTCCTTGCCAAGGAATAAGTGGCTTTAATCGTTTTAGAAATTCAGCAAATCCATTACAAGA
TCCAAAAAATAAACAGCTTGAAGTATTCATGAGCATTGTGGAGTTCTTGAAGCCAAGGTTCGTGTT
AATGGAAAATGTGGTGGACTTGCTTAGGTTTGCACATGGTTACCTTGGAAGATATGCACTAAGCAG
ACTTGTTGGAATGAACTACCAAGCACGGATGGGAATGATGGTTGCTGGGGCATATGGACTTCCACA
ATTTCGTATGCGTGTCTTCATGTGGGGTGCTCTTCCTTCAGAGAAATTGCCACAATATCCGTTGCC
CACACATAACGTTATTGTGAGGGGTGGCATTCCCACAGAATTTGAGTTAAATGCAGTAGATTTTGA
AGAAGGCCTGAAGGTCAAGCTAAAGAGAGAACTTCTTCTCGAGGATGCACTTTCAGATCTTCCTCC
TGTGGAAAATAATGAACCAAGGGATGAAATGCCATATATCGATGAGCCAAAATCAGTCTTTCAGCG
TTTCATAAGATCAAGGAGGGATGGTACGTTGGGTACTGTTTTGTATGATCATCGTCCCCTTCAGTT
AAACGAAGATGACTACCAGCGTGTAACTCAAATTCCCAAACAAAAGGGTGCAAACTTCAGGGACTT
GCCTGGGGTTCGTGTTCGTGCTGACAATGTTGTTGAATGGGATCCAGATATGGAAAGAGTAAAACT
TCCATCAGGGAAGCCTTTGGTTCCTGACTATGCAATGACTTTTGTTCGTGGCACTTCACAAAAGCC
ATTTGGTCGTTTATGGTGGGATGAAATTGTTTCAACAGTT***GTTACACGAGCAGAGCCCCATAATCA***
GGCTATATTACATCCAGTGCAGGACAGAGTGCTCACTATCCGTGAAAACGCAAGGCTGCAAGGTTT
CCCTGATTATTACAAATTGACTGGACCAATAAAAGAAAGGTACATACAAGTTGGAAATGCAGTTGC
AGTACCAGTTGCCCGGGCTTTAGGGTATTCTTTAGCATTGGCATTGAAAGGATTGTCGCGAGATCA
ACCATTACTTACATTACCACCTAATTTTCCATGTCTTGAGGAACTGGTCTCCAATGACGAATCTCT
AGATAAGGTTTAATTATCTGAACGTTACGT***CAAGGTACTAACCTCAACTGATTATTCCAAGTATTA***
TCATTCATTAGTTTACTGAAAACAGTTGTTGCAATGCAAGTTCTGCATTAACATTATATTTTTTTG
TTAGTTGCTAATTTAGGACAATGGAGAAGTTGGAAGTAGGTATTGTGTTTTTGTAGGTATCATTAA
TTTCTACATTGGATTGACAACTTAACTTATTTTGGTGCAAATGATAACTGTTGTATTGCAATGGTT
AAAAAAAAAAAAAAAAAAAAAAAAAAAAAAAAAAAAAAAAAAAAAAAAAGAAAAAAAAAAAAAAAA
AAAAAAAAAGTACTCTGCGTTGATACCACTGCTTGCCCTATAGTGAGTCGTATTAGAAGGGCAATT
CGTTTA

d

SlCMT4 (Sequences highlighted RED was cloned into PVX/SlCMT4)

TCAACGCAGAGTACATGGGATGACCCAAAGCGCGTATTCTATTCTACTTTGGAGAATGATAATTTA
CTAGACTGCATAGTTTCCAAAGTTAACGTGGTAGAATTACCAACCAGGCATGATTTGAATAAAAAG
GATGTTCCACCTGCTCACTTTTATTACGACATGGAATACTGTGTTGATTATTCCACATTCCGTACG
TTGCATAATGTCAAAAGCTCTGTCTCCCCTTCGCTAGTTGATGTGTCATATAAGCCTATCACTACA
TATCCTCTGGAGGTATCACCAAGTTGTGAACCTATGAAAGTGGAGTTGTCATTATTGGATCTATAT
GCTGGTTGTGGCGGCATGTCAATGGGATTGTGCCTTGGCACAAAACTCTCTGGTCTTAATCTTGTG
ACGAAATGGGCAGTTGATTTTAATAAAGCTGCCTGTGATAGCCTGAAACTGAATCATCCTCAGACA
CATGTTAGGAATGAAGGTGTTGAGAATTTTCTAGAGCTACTGAAAAGATGGGAGAAGCTAATTAAG
TCGTACGGCTGTAGTGATATCAAAACAAGCTCTAATATTGAGCTAGACGACAGAGATGAAGGCGAA
AACAATGATGATTCTCAGTCTGGTTCCAATGCATCTTCTGGAGAATATGAGGTATTAAGATTCGTT
GACATATGTTACGGGGATCCCAACAATGACGGAAAATCTGGACTTCATTTT***AAGGTGCGATGGAAA***
GGTTATGGTCCGAGTGAAGATACTTGGGAGCCAATTGAGAACTTGAAAAATTGCGGAGACAGTATA
AAAGATTTTGTTCGAAGGGGGCAACAGTTGAAAATTCTGCCTCTACCAGGTGACGTTGATATGATA
TGTGGGGGTCCTCCTTGCCAGGGCATCAGTGGATATAATCGCCACAGAAATACTGATGACCCATTG
TCTGATGAAAAAAATCGTCAAATTATTATATTTATGGATGTTGTGGAATTCCTACGGCCTAAATAT
GTGTTGATGGAAAATGTTGCTGACATATTGAGGTTCGATAAAGCATCTCTTGGAAGATATGCTTTA
AGCCGTTTGGTACATATGAGATACCAAGCAAGACTTGGAACTATGGCAGCTGGTTGTTATGGTCTT

CCACAATTCCGATTGCGAGTTTTCTTTTGGGGTGCACTTCCAAGTGAGAGGTTGCCCCCATTTCCA
CTTCCTTCGCATGACGTGATTGTTAAATATTGGCCCTCACCTGAATTTGAGCGTAATACTGTTGCT
TATGAGGAAGGACAACCACGTGACCTTGAGGAAGCTCTTGTTCTTCGTGATGCGATATCTGATATG
CCAGCTGTCACATGGCATGAAACTCGTGAAGAAAGGCCATATGAGATGCCCCCTGAAACAGAGTTC
CAGAAATACATAAGATTATCTAAACATGAGATACTGAGTTGTACATCAACTGGAGTCAAGGAAACA
AAAGAACCTGTTTTGTCTGATCATCGGCCTTGCCAACTAAACGAGGACGATTACTTGCGAGTTTGT
CTAGTCCCACGTAGAAAGGGAGCAAATTTCAGGGACCTCCCTGGTGTAATTGTAGGAGGGGATAAT
GTGGCTCGTCGTGATACTAAAGATCCAAAGGTTCTACCAAACGGAAAGCCCATGGTTCCTGACTGT
GCCTTTAACTTTGAGCATGGAAAGTCTAAGAGGCCATTTGCAAGGTTGTGGTGGGATGAAACAGTA
GCAACATTAGTGACGTTTCCTAATCATCGCGCCCAGGCTATCTTACACCCAGAGCAAGATCGAGTA
CTGACCATACGAGAGTATGCAAGATTGCAAGGTTTTCCAGATTTCTATAGGTTTACTGGTACTCTC
AAGGAAAGATATTGCCAAGTTGGGAATGCTGTAGCAGTTCCTGTTGGTCGCGCACTTGGGTACGCG
CTTGGACTTGCCTATCAGAGGCTAGCTGGAAATGAGCCTCTTATTAAATTGCCATCAAATTTCTCA
TTCTTAACACCTCCAATTGATGACATTGTTGTCTTGCAAACATAAGCTCATATTCATCATTTTTTT
CCTCAATTGGCGATTTCGGAGCTACTGCTGATACTTAGCGGATTACTCCATTTTTCTAGTATGAAC
TTCTATTTAACAGAACAGATACTAGACTGGGGCAGCCAAGTTGATTAATTTATTGATCAATGAACT
AGCATTCCTTTAAAAAAAAAAAAAAAAAAAAAAAGTACTCTGCGTTGATACCACTGCTTGCCCTATA
GTGAGTCGTATTAGAAGGGCAATTCGTTTAAACCTGCAGGACTAGTCCCTTTAGA

e
SlDRM5
GGCACCAGCTTCCTTCGCTCTCAAATCTATGATATTAAGAAGATTTATGATATAGCGCAGCACGGA
GTTGCTTGACACCCTTTGATCGACAAGGATTCTCAGGGTGCTCAATTACTCTTCGTGCTGAACACT
TTAGAGTTCTTTTCAGGCTTACATTGTGAAATGGACAAACATCTTTCTGAAGAAGATAGTGACAAC
ATTGACTGGGATACTGAAGATGAGTTAGAAATACAGGATACAACATTTTCCTCATGCAGGGATTTA
AGAACTAATGGACAGTATGCTATTAGTGGTGATGGGGAGGCAAGCTCATCATCAGTACCTGGCCAG
TCTACGTTCATTCAGAAGTTTTTAGTGATGGGATTTTCTGAAGAGTCCATTGCAAAAGCAATAGAG
CAAAATGGAGAAAATTCAGATTTGGTGCTGGATGCTCTTTTGACATTAAAGGCCATCGAAGATTCT
CCTGAAGAACAGCCCAGTGCTAGCCCTCACCTGGAGCCCTGCATTAATTCTGATGATAGCTCTTCT
GAATACAATGAGAACTTTCTGGATGATGTTTATGATGAAGATAGTTGGTCCTCTGACTCAGACTAC
TGTACAAATTCTGTTAAACAGTGCTATGTGAAAGAAGAGAGCAATTCTTTGTCTGAAAAAGAGCAG
ACAATCTTATTTCTGGCAAATATGGGATACCCAGTGGAAGAGGTTTCTATAGCAATGGAGAGATGT
GGTCCAGAAGCATCGGTTTCTGAATTGACAGATTTCATTTGTGCTGCTCAAATGGCAAGAGAAGAA
GATCCCTATTTGCCAGAAGATGTAAAGCCAAAACTGAACCATGGTAGTGGTGGATACAAGAAGAGG
AAGATGTTCAATCAGTTGTGCAAAAGCAAAAAGCCAAGGGCGATTTTTGATGAAGAGACAATTCGT
TTGCCCAAACCTATGATTGGATTTGGGGTTCCTACAGAATCAGTTTCTGCAATTGTCCGAAGAACT
ATTCCGGAGCAAGCTTTTGGCCCCCCTTTTTTCTATTACGAAAATGTTGCTCTAGCTCCAAAGGGT
GTGTGGGATACCATTTCCAGATTCCTATATGATATTGAGCCTGAGTTTGTCGATTCAAAGTATTTT
TGTGCTACTGCAAGAAAAAGGGGTTATATTCATAACCTACCTACTGAAAATAGATTTCCCTTGCTT
CCACTTCCTCCACGCACCATTAATGAGGCACTTCCCCTTACAAAGAAATGGTGGCCATCTTGGGAT
CCACGGACAAAGTTAAATTGTTTGCAAACAGCTATTGGAAGTGCAAGATTGACTGATAGGATCAGG
AAAGCTGTGGAGGCCTTTGACGGTGAGCCACCTATGAGGGTGCAAAAGTTTGTTCTTGATCAATGT
CGGAAGTGGAATTTGGTGTGGGTTGGGAGAAACAAAGTTGCTCCTTGGAGCCAGAGAATTGGAAAT
GCTATTGGGGTTCCAAAGAACCATACCAGGGGAGGTGGTATAAGTAGGACCGATAGATACAAATCG

CTTGGTAACTCATTCCAGGTTGACACAGTGGCATACCATTTATCAGTGTTGAAAGACATGTTTCCA
AATGGGATGAACGTCTTATCACTCTTCTCTGGGATTGGCGGTGCTGAAGTTGCTCTTTACCGTCTT
GGGATTCAACTAAACAATGTGGTCTCTGTGGAAAAATCTGAAGTGAACAGAAACATTGTGAGAAGT
TGGTGGGAGCAAACTAATCAGAGAGGCAATCTTATTGATTTTGATGATGTGCAGCAGTTGAATGGA
GACCGCTTAGAACAACTGATAGATTCATGTGGCGGATTTGATTTACTGATTGGTGGAAGCCCGTGC
AACAATCTTGCAGGCAGTAACAGGGTGAGCAGGGACGGTCTTGAAGGCAAAGAATCGTCTCTATTT
TATGACTATGTTCGGATACTGGACTTAGTCAAGTCCATAATGTCTAGACAGAGATAGTATGACAGA
AATTCGTTCTAGCTTCAGATTTTTAGTTATCTTTCAAAATTATGAAACATTACATTAAGGGATAAA
GCCATCACATAAAATTTCTGGCTTAAGATTCTACATCGACTGGTCTCCTACAATACCAGTTGATGA
GCATTCTATTTCGCTTTGAAGCGAAAACAAACATGAAGTGTAAAGTACATTGTTACTATTAGTTAT
TGGCCTAACATGCCATTTCAGTTGATGAGCATGCACAGTTACAGTGTTGTATCCTCCATGAATAAT
ATTCTGGAATTTGCATAAGAAAGCTCTGCACTTGAATCTCAAAAAAAAAAAAAAAAAA

f

SlDRM6

GTCCAGTGTTGGAAGCGTAAAACTCCAAATATGGGAATCTCTCTTATGCTTTAAAACCAAACAAGA
ATCTCTTCTTCGTCAATTTTCTCTAAAATCTATGGATATCTGATATAGATCAAAATTGATTGTTGC
ATTGATCACTGAAAAATTTCAGGGTGCTGATTTATTCTTCTTGCTGAACAAATTGGAGTTCTTTTT
CAGGCTTACCTTGAGAAATGGACAAAAATCTTTCTGGAGAAGATAATGATGACATTGACTGGGATA
CTGAAGATGAACTTGAAATACAAGAAATACAGGACACAGTGTTTTCCTCATGCACGGATTTAAGAA
CTACTGGGCAGCATGTTGTTTGTTGCGATGTGGAGGCAAGCTCGTCATCAGTACCCTTCCGGTCCA
AGTTCATTCAGCAGTTTGTAGTGATGGGATTTCCTGAAGAATCTATTGCAAAAGCAATAGAGCAAA
ACGGAGAAAATTCAGATTTGGTGCTGGATTCTCTTTTGACTTTTAAGGCCCTTGATGACTCTCCTG
AAGAACAGCCCAGTGTTAGCCCTCCGCTGGAACCCTCCATTAGTTCTGATGACAGCGCTTCTGAAT
ACAACAAGATTGTTTTGGATAATGTTTATGAGGATGATAGTTGGTCTTCTGACTCAGACAACTACA
TAAATACTGTTAAGCAGTGCTACTTGAATGACGAGGGAAGTTCTTTGTCTGAAAAAGAAAAGATGT
TATTGTTCCTGGGAAATATGGGATATCCAGCGGAAGAGGCCTCCATAGCAATGGATAGATGTGGTC
CAAAAGCATCACTCCCTGAATTGGTAGATTTTATTTGTGCTGCTCAAATGTCAAGAGCGGAAGATC
CCTATCTCCTAGAAGATGTGAAGCCAAACCTGAAGGATATATAGAAAGAATGAAGAGGATACAAAA
AGAGGAAAATGTACAATGAATTGTGCAAACGGAAAAAGCAAAGGGAGATTTCTGTTGAAGAGCCAA
TTCGATTGCCCAAGCCGATGATTGGCTTTGGAATTCCTACCGAATCAGTTCCAAGAATGGTTCAAC
GAATTCTTCCCGAGAAAATCATTGGCCCGCCTTATTTCTATTATGAAAATGTCGCCCTGGCTCCAA
AGGGTGTGTGGGACACCATTAAGAGGCATTTGTATGAGATTGAGCCCGAGTTTGTTGACTCGAAGT
ACTTTTCTGCAACTGCAAGAAAAAGAGGGTATCTTCATAATCTGCCGATTGAAAACAGATTTCCTT
TGTTTCCACTTCCCCCACGGACCATTCATGAGGCACTTCCCCTTTCGAAGAGATGGTGGCCATCTT
GGGATACTAGATCAAAGTTGAACTGCTTACAAACAGCCATTGGGAGTGCAAGGTTGACGGATAAGA
TCAGGAAAGCTGTCGAGAAGTATGATGGTGAACCACCTATGGAAATACAGAAGTATGTACTCTATC
ATTGCAAGAAGTGGAATTTGGTCTGGGTAGGAAGAAACAAAGTTGCCCCTTTGGAACCTGATGAAG
TTGAAATGCTATTGGGGTTCCCAAAGAATCACACAAGGGGAGGAGGTATAAGTAGGACGGACAGAT
ACAAGTCACTCGGTAACTCTTTCCAGGTTGACACAGTGGCGTACCACTTATCAGTGTTGAGAGACT
TGTTTCCAAATGGAATCAATGTCTTATCACTCTTCTCTGGCATTGGTGGTGCTGAAGTTGCTCTCT
ACCGTCTCGGTGTTCCCCTCAACAATGTTGTTTCAGTTGAAAAGTCTGAAGTGAACAGGAATATTG
TGAGAAGCTGGTGGGAGCAAACAAATCAGAGGGGGAATCTCATACATTTTGACGATGTGCAGCTGC
TGAGCAGAGATCGGTTGAAGAAGTTAATAGAATCAGTTGGTGGATTTGATTTAGTGATTGGTGGAA

GCCCATGCAACAACCTTGCAGGCAGTAACAGAGTGAGCAGGGATGGGCTTGAAGGCAAAGAGTCTT
CTCTGTTTTTTGATTATGTTCGGATATTGGATGATGTCAAGTCCATAATGTCTAGACATAGATGAG
ATACTCTCTCTCATTTAGATATCCTATTCCTTTTTCTCAACCTTGCCAAGGCTTAAGGTTATTCTT
AATTAGTAGGGTGAAATCAAACTAGATACTAATTGCAATTATGAAACTGATGAAATTTTGGTGGTT
GATGAAGTAATGTTATTGTTTCTGGTTCAGTTTTTCCAGAACCCAGGTATCT

g

SlDRM7 (Sequences highlighted RED was cloned into PVX/SlDRM7)

GGTAGCTAAGTATATCTCCGATAACTAAATTTTGGGTGATGATTTATTTTTGTTGCTGCAGAGTTC
GGAGTTCTTTTTAAGGAATTACCTTTAAAAATGGATAACAACTTTTCTGGAGAAGACAATGACAGC
ATTGACTGGGATACTGAAGATGAATTAGAAATACAAGAAATGCCGGATGCAACATTTTCCTCGTGC
ACCAATTTAAGAAGTGTTGGATATCATACCGTTAGTGGTCATAGGGAGGCAAGATCATCATCAGAA
CCATGTCAATCTAAGTTCATTCAACAATTTATCGTGATGGGATTTCCTGAAGAGTCTATTGCAAAA
GCGATAGAGCAAAATGGAGAAAATGAAGGTTTGGTGCTAGATGCTCTTTTGACATTCAAGGCACTT
GAAGATTCTCCTGAAGAACAGCCGAGTACGAGCACTCAGATGGAACCCTGCATTACTTCTGATGAT
AGCTCTTCCCAGTACAACGAAAACTTTTTGGATGATGTTTCTGAAGATGATAGCTGGTCCTTGGAC
TCGGACAATTGTGTAAATTCTGCTAAACAGAGCTACTTGAATGACGACAACTGTTCTTTGTCTGAA
AACGAGAAGACATTATTATTCCTAGCAAATATGGGATACCCTGCGGAGGAGGCTTCCATCGCAATG
GAGAGATGTGGTCCAGAAGCACCGTTTCCGGAGTTGATAGATTTCATGTGTGCTGCTCAAATGGCA
AGAGAAGAAGACGTCCATCTGCCCGAAGATGAAAAGCCAAAACTAAATAGTGGTGGATACAAAAGG
AAGATGTATAATGAAGTTCGCGTAAAGAAAAAGCAAAGGGCGATAACTGATGAAGAGACAATTCAT
TTGCCCAGACCTATGATTGGATTCGGGGTTCCTACGGAATCCTTACCAGCTGTTGTGAAAAGAACT
CTGCCAGAGCAAGCTATTGGCCCCCCTTTTTTCTATTATGAAAATGTTGCTCTTGCTCCGAAGGGT
GTGTGGGACACTATGACAAGATTCCTTTACGATATTGAGCCCGAGTTTGTTGACTCGAAATATTTT
TGCGCTACCGCAAGGAAAAGAGGATATATTCATAATCTACCAATTGAAGATAGATTTCCTTTACTT
CCACTTCCTCCACGTACCATTCACGAGGCGTTTCCTCTAACGAAGAAATGGTGGCCATCTTGGGAT
ACACGAACAAAGTTAAATTGCTTACAAACATCTATTGGGAGTGCAAGGTTAGCTGATAGAATTAGG
AAAGCTATGAAAGCGATGGAGAACTTCGATAGTGAGCCACCGTTGATGGTGCAAAAGTATGTTCTA
GACGAATGCCGGAAATGGAATTTGCTGTGGGTTGGTAGAAacaATAAAGTCTCTCCTTTGGAGACT
GATGAATTTGAAATGCTAATGGGATTTCCACGGAATCATACCCGTGGAGGAGGTATAAGTAGGACC
GATAGATACAAGTCGCTTGGTAACTCGTTCCAGGTCGACACAGTGGCATACCATTTATCGGTGTTG
AAAGACTTGTATCCAAATGGTATAAATGTTTTATCACTATTTTCTGGAATTGGCGGTGCTGAAGTT
GCTCTGTATCGTCTCGGTATTCCATTGAACAATGTGGTTGCCGTAGAGATATCTGAAGTTAACAGG
AATATTCTGAGAAGCTGGTGGGAGCAAACAAATCAAAAGGGGAATCTTATAGATTTCCATGATATC
CAGCAGCTGAACGGAGACGTCTTGGAGCAAATGATCGATTCAATTGGAGGATTTGATTTAGTAATT
GGCGGAAGCCCCTGCAACAACCTCACTGGAAGTAACAGGGTGACTAGAGACGGACTCGAAGGTAAA
GATTCTTCTCTATTTTATGAC***TATGTTCGGGTAGTGGACTTGGTCAAATCCATTATGTCTAATCGT***
AGAGTGTGACGAAAATATGTTTTGAGTTTCTGAACTTGGAAGGCAACAAGTAAAAAGTTGTCTTTG
ATTTTGGTTTGTACATATTTTTCGTAAAAGGGGGCACAAAGTAACTCTCTTAGTGCCATCCTAATA
TAACTGTTGAACGTTCGTGGAAAGAATGAACTGTTGAACTTCTCATTGTAAACGACATTAAATGAT
GTTGAAAATGAACTTGTGATTTTCGCATAAAAAAAAAAAAAAAAAAAAAAAAAAAAGTACTCTGCGT
TGATACCACTGCTTGCCCTATAGTGAGTCGTATTAGAAGGGCAATTCGTTTAAACCTGCAGGACTA
G

h

SlDRM8

GATATTTTGTTTTCATCAGCTGAATTAAGCTCTCTCAGCCTGAATTTCATCTGAGCTCCGATCAGT
TTTCGCCGCAGTTAAAGGCTCAAGGAAAGCACTGTTTTATGCTTTTGCTGTGGCACATAAGAGACA
ATTCTCTAGCTTCGCCCTGTCCAATTTCACATTAAAATGCGTGAATATCCAGATAGTGAAAACTCT
TCAAAGCCTGAAGGTGTGAGTGGAATCATGCCTAAAGTTGAAGATCCATATTCCGAATTTCCATCT
CTGTACACATATACAATGCCTATGGGGGACAATATTGCAAGCTCATCAGGCAGCAATGTGAGGTCA
TCTCTTCTGACCATGGGTTTTAAGGCATCACTTGTTGATAAAGCAATTGAGGAAAAGGGTGAAGAC
AACATTGACTTGTTACTGGAGACTCTTTTTGCAAAATCTGATCCTCCTAGAGCAGAATCATCAGAT
TCTCTGGATAGTTTGTTTTGTGATGACGAGGACATAAACAGTTCTGCCAAGTATGATGGAGATGCA
CATATAAAAGAGGTGAAGCTGCTCCTGTCAATGAACTAGTGGATGTAATCTTTGCAGCTCGAATCG
CTGGGAATTACAAAAAAGATGATGATGATGATGTTTCTGTTGTTGAGATTAAAGAAAGGAACAAGG
AATGCACAACTGAGTCTCTGTTTGGAACCATGGAGAAGACATTGAAATTGCTTGAAATGGGCTTCT
CTGAGAATGAAGTTTCCACAGTTATTGAGAAGTTTGGTTCTGAAGTTCCTCTCGAAGAGCTTGCAA
ATCTGATTATAGATCCTAGTAGTAGAAGAAGAATGGACAAGCATCTCTTAAACTCTCTCGGCAGGA
ATGTTTCAATTGGTTTTAATCCAGTAGCTGTTAAGAAAGAGGAATATAGTGTGGATACCTCTGAAT
CTAGAGAACTTGACCTACTGGAAAAGCTTAAAGGGAAACGGCCAAAGGAGAATTACATTGATGAGA
TAGACACTAAGAGACCGAAATCAGAGTATGATGAAGCCTTTAACAATTCCCTTGGTCCTTCATGGC
AAGAAATATTAGGCATCAATACCACCAGGCCTTATAAAGTTCATCGAAGAGCGATTCACCAAAGAC
TAAGAGTTCTGGATTGCCATGACACACAGAAATTATCTATGCCGAATTCATGCAGGAGCCTTGATA
AGATGGTGGCTAAGGCTCCCTATTTCTTTTATGGGAATGTAATGAACTTATCTCATGACTCTTGGG
TAAGAATATCACAGTTTTTGTATGCCATTGAGCCAGAATTTGTTCATACTCAACTTTTCTCAGCTC
TGAGTCGTAAAGAAGGTTATGTACATAATCTTCCTAGTGAAAATCGATTTCACATTGTTCCGAAAC
CACCAATGACGATTCAAGAAGCAGTTCCCAGTAGCAAAAAATGGTGGCCATCATGGGATACAAGGA
AACACTTGAACGGCATCAATTCTGAGACATCTGTTGTATCTCAGCTATGTGATAGGCTTGAAAGAA
CAGTGAGTGATGCCCAGGGGTTTCCTTCAGTTGACAGACAGAGAGATATCCTCAACCAGTCCCAGA
TATTTAATCTAGTCTGGGTTGGCCGCTACAAATTGGCAGCTGTAGGGCCAGAACAAATAGAACGTA
TTCTGGGCTACCCAGAAAATCACACTCGAGTTGCTGCATTTAGCTTGATGGAAAGACTTCTATCTC
TGAAACATTGCTTCCAGGTAGATACATTGGCTTATTGTCTCTCTGGATTGAAGCATTTGTACCCTG
GAGGGTTGACTGTGTTGTCAATCTATAGTGGTATTGGTGGAGCAGAAGTTGCATTGCATCGCCTTG
GTATTCGCTTAAAAGCTGTTGTCTCAATAGAGGCTTCTGAGAAAAACCGGAGAATTCTCAAGCAAT
GGTGGAGTAGTTCAGGACAAACAGGAGAACTCGTGCAGATGGAAGACATCCACAAGTTGGCCAGTA
ACAAGGTAGAGGTCTTGATTAACAACTTTGGTGGTTTTGATTTCATCATATGCCAGAACCCATGTA
CATACTCTTCTAAAGGTAATTTAGCTGCAGATATGGATAGTCATGCAAGTTTAGATTTCATGTTGT
TTCATGAGTTTGTTCGTGTCCTCCAACGTGTAAGAAGCACAATGGGGCGGAATTAGCAAGTTCCTT
AGCTTTAATCTGTAGGCACTTAGTGAACTGAGCTTAATGTTATGTTGTCCTGGATTATGACTTCTA
CTTTGTAATATATTAAGCTTATATTGTGAATGGTTCCCATTACATTATTGTAAAAAAAAAAAAAAA
A

i

SlDRM9

TAGCGAAAAAGTTTCCTCTGTTAAAGCAAAAAACGGAGAGGACTTTCTCGTCCAGTGTTGGAAGCG
TAAAACTCCAAATATGGGAATCTCTCTTATGCTTTAAAACCAAACAAGAATCTCTTCTTCGTCAAT
TTTCTCTAAAATCTATGGATATCTGATATAGATCAAAATTGATTGTTGCATTGATCACTGAAAAAT

TTCAGGGTGCTGATTTATTCTTCTTGCTGAACAAATTGGAGTTCTTTTTCAGGCTTACCTTGAGAA
ATGGACAAAAATCTTTCTGGAGAAGATAATGATGACATTGACTGGGATACTGAAGATGAACTTGAA
ATACAAGAAATACAGGACACAGTGTTTTCCTCATGCACGGATTTAAGAACTACTGGGCAGCATGTT
GTTTGTTGCGATGTGGAGGCAAGCTCGTCATCAGTACCCTTCCGGTCCAAGTTCATTCAGCAGTTT
GTAGTGATGGGATTTCCTGAAGAATCTATTGCAAAAGCAATAGAGCAAAACGGAGAAAATTCAGAT
TTGGTGCTGGATTCTCTTTTGACTTTTAAGGCCCTTGATGACTCTCCTGAAGAACAGCCCAGTGTT
AGCCCTCCGCTGGAACCCTCCATTAGTTCTGATGACAGCGCTTCTGAATACAACAAGATTGTTTTG
GATAATGTTTATGAGGATGATAGTTGGTCTTCTGACTCAGACAACTACATAAATACTGTTAAGCAG
TGCTACTTGAATGACGAGGGAAGTTCTTTGTCTGAAAAAGAAAAGATGTTATTGTTCCTGGGAAAT
ATGGGATATCCAGCGGAAGAGGCCTCCATAGCAATGGATAGATGTGGTCCAAAAGCATCACTCCCT
GAATTGGTAGATTTTATTTGTGCTGCTCAAATGTCAAGAGCGGAAGATCCCTATCTCCTAGAAGAT
GTGAAGCCAAACCTGAAGGATATATTGAATGATTGTGGTGGATACAAAAAGAGGAAAATGTACAAT
GAATTGTGCAAACGGAAAAAGCAAAGGGAGATTTCTGTTGAAGAGCCAATTCGATTGCCCAAGCCG
ATGATTGGCTTTGGAATTCCTACCGAATCAGTTCCAAGAATGGTTCAACGAATTCTTCCCGAGAAA
ATCATTGGCCCGCCTTATTTCTATTATGAAAATGTCGCCCTGGCTCCAAAGGGTGTGTGGGACACC
ATTAAGAGGCATTTGTATGAGATTGAGCCCGAGTTTGTTGACTCGAAGTACTTTTCTGCAACTGCA
AGAAAAAGAGGGTATCTTCATAATCTGCCGATTGAAAACAGATTTCCTTTGTTTCCACTTCCCCCA
CGGACCATTCATGAGGCACTTCCCCTTTCGAAGAGATGGTGGCCATCTTGGGATACTAGATCAAAG
TTGAACTGCTTACAAACAGCCATTGGGAGTGCAAGGTTGACGGATAAGATCAGGAAAGCTGTCGAG
AAGTATGATGGTGAACCACCTATGGAAATACAGAAGTATGTACTCTATCATTGCAAGAAGTGGAAT
TTGGTCTGGGTAGGAAGAAACAAAGTTGCCCCTTTGGAACCTGATGAAGTTGAAATGCTATTGGGG
TTCCCAAAGAATCACACAAGGGGAGGAGGTATAAGTAGGACGGACAGATACAAGTCACTCGGTAAC
TCTTTCCAGGTTGACACAGTGGCGTACCACTTATCAGTGTTGAGAGACTTGTTTCCAAATGGAATC
AATGTCTTATCACTCTTCTCTGGCATTGGTGGTGCTGAAGTTGCTCTCTACCGTCTCGGTGTTCCC
CTCAACAATGTTGTTTCAGTTGAAAAGTCTGAAGTGAACAGGAATATTGTGAGAAGCTGGTGGGAG
CAAACAAATCAGAGGGGGAATCTCATACATTTTGACGATGTGCAGCTGCTGAGCAGAGATCGGTTG
AAGAAGTTAATAGAATCAGTTGGTGGATTTGATTTAGTGATTGGTGGAAGCCCATGCAACAACCTT
GCAGGCAGTAACAGGGTGAGCAGGGATGGGCTTGAAGGCAAAGAGTCTTCTCTGTTTTTTGATTAT
GTTCGGATATTGGATGATGTCAAGTCCATAATGTCTAGACATAGATGAGATACTCTCTCTGATTTA
GATATCCTATTCCTTTTTCTCAACCTTGCCAAGGCTTAAGGTTATTCTTAATTAGTAGGGTGAAAT
CAAACTAGATACTAATTGCAATTATGAAACTGATGAAATTTTGGTGGTTGATGAAGTAATGTTATT
GTTTCTGGTTCAGTTTTTCCAGAACCAGGTATCTAAAGAATATATAAGAGTATACCAAAAAAAAAA
AAAAAAAAAAAAAAA
